# Phenotypic and Genotypic Characterization of Recently Isolated Multidrug-Resistant *Acinetobacter baumannii* Clinical and Aquatic Strains and Demonstration of Silver Nanoparticle Potency

**DOI:** 10.3390/microorganisms11102439

**Published:** 2023-09-28

**Authors:** Irina Gheorghe-Barbu, Viorica Maria Corbu, Corneliu Ovidiu Vrancianu, Ioana Cristina Marinas, Marcela Popa, Andreea Ștefania Dumbravă, Mihai Niță-Lazăr, Ionut Pecete, Andrei Alexandru Muntean, Mircea Ioan Popa, Liliana Marinescu, Denisa Ficai, Anton Ficai, Ilda Czobor Barbu

**Affiliations:** 1 Faculty of Biology, University of Bucharest, Intr. Portocalelor No. 1–3, 060101 Bucharest, Romania; irina.gheorghe@bio.unibuc.ro (I.G.-B.); ovidiu.vrancianu@yahoo.com (C.O.V.); ioana.cristina.marinas@gmail.com (I.C.M.); bmarcelica@yahoo.com (M.P.); andreeadum29@gmail.com (A.Ș.D.); ilda.barbu@bio.unibuc.ro (I.C.B.); 2 The Research Institute of the University of Bucharest (ICUB), B.P Hasdeu No. 7, 050095 Bucharest, Romania; 3 National Institute of Research and Development for Biological Sciences, 296 Splaiul Independentei, District 6, 060031 Bucharest, Romania; 4 National Research and Development Institute for Industrial Ecology (INCD ECOIND), 050663 Bucharest, Romania; mihai.nita@incdecoind.ro; 5 Central Reference Synevo-Medicover Laboratory, 021408 Bucharest, Romania; pecete.ionut@yahoo.ro; 6 Cantacuzino National Medical Military Institute for Research and Development, 050096 Bucharest, Romania; muntean.alex@gmail.com (A.A.M.); mircea.ioan.popa@gmail.com (M.I.P.); 7 Department of Microbiology II, Faculty of Medicine, Carol Davila University of Medicine and Pharmacy, 020021 Bucharest, Romania; 8 Faculty of Applied Chemistry and Materials Science, University Politechnica of Bucharest, Gh. Polizu, No. 1–7, 011061 Bucharest, Romania; liliana10marinescu@gmail.com (L.M.); denisaficai@yahoo.ro (D.F.); anton_ficai81@yahoo.com (A.F.)

**Keywords:** multidrug resistance, virulent strains, wastewater, surface water environments, silver nanoparticles

## Abstract

This study aims to demonstrate the effectiveness of silver nanoparticles (Ag NPs) on multidrug-resistant (MDR) *Acinetobacter baumannii* (AB) strains isolated from the clinical and aquatic environment. Three types of Ag NPs were investigated for their antimicrobial, antibiofilm, and antivirulence properties on a total number of 132 AB strains isolated in the same temporal sequence from intra-hospital infections (IHIs), wastewater (WW), and surface water (SW) samples between 2019 and 2022 from different Romanian locations and characterized at the phenotypic and genotypic levels. The comparative analysis of the antimicrobial resistance (AR) profiles according to the isolation source and the geographical location demonstrated a decrease in MDR level in AB recovered from WW samples in 2022 from north-eastern/central/southern regions (N-E/C-W/analyzed strains S): 87.5/60/32.5%. The AB strains were lecithinase, caseinase, amylase, and lipase producers, had variable biofilm formation ability, and belonged to six genotypes associated with the presence of different virulence genes (*ompA*, *csuE*, *bap*, and *bfmS*). The Ag NPs synthesized with the solvothermal method exhibited an inhibitory effect on microbial growth, the adherence capacity to the inert substratum, and on the production of soluble virulence factors. We report here the first description of a powerful antibacterial agent against MDR AB strains circulating between hospitals and anthropically polluted water in Romania.

## 1. Introduction

*Acinetobacter* species are widely distributed in various environments, including soil, water, sewage, humans, food, and animals. Among these species, *Acinetobacter baumannii* (AB) stands out for its remarkable resilience and role as a potent pathogen [1]. Usually, AB is encountered mainly in moist tissue infections, such as mucous membranes or skin areas resulting from wounds or injuries [2]. It is notorious for being a formidable opportunistic nosocomial pathogen, mainly affecting immunocompromised patients. Its significance is even more pronounced during healthcare crises like the COVID-19 pandemic due to its high levels of resistance to multiple drugs, including extended-drug-resistant (XDR) and pandrug-resistant (PDR) phenotypes [3]. This resistance profile presents a significant challenge, often leading to treatment failures in affected patients.

In recent years, the prevalence of multidrug-resistant (MDR) AB strains has escalated considerably in healthcare environments. These strains have acquired multiple resistance mechanisms against antibiotics, including carbapenems and colistin, as well as resistance to disinfectants and the presence of virulence factors (VMs). In particular, Romania has experienced high MDR rates in clinical AB isolates. In 2021, the highest resistance levels were observed for fluoroquinolones, carbapenems, and aminoglycosides [4]. Resistance to β-lactam antibiotics (encompassing penicillins, cephalosporins, carbapenems, monobactams, and β-lactamase inhibitors) in AB is a consequence of various factors, such as the production of enzymes that modify antibiotics, efflux pumps, changes in porin proteins, protection of antibiotic targets, and the formation of biofilms. However, the primary mechanism behind β-lactam resistance in AB involves the production of Ambler class D serine-β-lactamases. These β-lactamases fall into several categories, including OXA-23, OXA-24/40, OXA-58, OXA-143, and OXA-235 types [5]. In addition to antimicrobial resistance (AR), AB’s ability to cause disease is further augmented by various virulence factors (VMs). These VMs include outer membrane proteins like OmpA, porins such as CarO, pili structures, capsular polysaccharides, the siderophore acinetobactin, and the formation of biofilms [5,6]. Although, until recently, the *A. baumannii* was considered a low virulence microorganism responsible mainly for infections affecting mainly immunosuppressed patient, its rapid development of broad-spectrum AR is considered as being of great interest to the medical community [7,8]. Consequently, there is an immediate and imperative need to enhance the effectiveness of antimicrobial treatments against these highly virulent bacterial strains.

**Figure 1:**
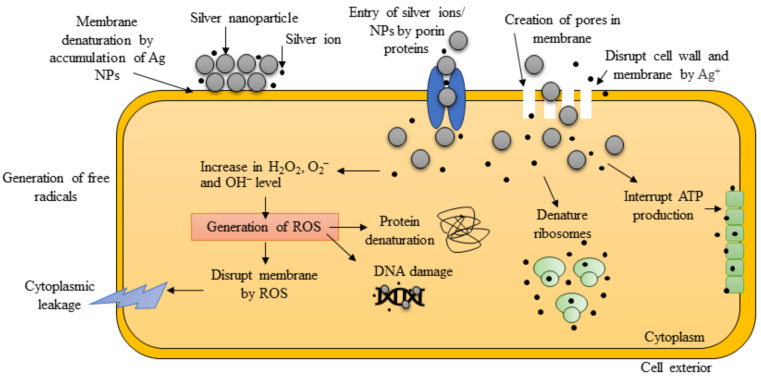
Graphic representation of the antibacterial mechanism of action of Ag NPs (created with Biorender.com (accessed on 19 September 2023)).

In the last few years, interest has increased in applying nanoparticles (NPs) as therapeutic regimens [9,10]. Specifically, the scientific community has shown significant interest in silver nanoparticles’ antiseptic and antimicrobial characteristics (Ag NPs) against Gram-positive and Gram-negative bacteria [11]. The production of Ag NPs considers factors such as pH, temperature, choice of media, solvent type, and the preparation method. These Ag NPs, characterized by their low ecological toxicity and substantial surface capacity, can impede the accumulation of biofilm materials responsible for evading and protection mechanisms and growth of the MDR AB strains [12]. The relevance of Ag NPs has been demonstrated especially in the biomedical field due to their antibacterial, antifungal, antiviral, anti-inflammatory, anti-cancer, and anti-angiogenic, and anti-oxidative properties. Ag NPs accumulate on the cell wall and membrane, causing cytoplasm shrinkage and membrane disruption. More than that, Ag NPs can bind to membrane proteins and affect membrane permeability, the respiration chain, and ion transport [13]; Ag NPs can enter the cell where they can interact with DNA and/or with intracellular proteins, thus interfering with transcription, translation, and sugar metabolism [14] (Figure 1).

Recent studies reported the Ag NPs’ efficiency in the biogenic formulation of new antimicrobial agents and drug delivery compounds. In two recent studies, Yassin and collaborators aimed to demonstrate the efficiency of Ag NPs synthesized using a green approach based on *Origanum majorana* and *Salvia officinalis* extracts on MDR strains. The disc-diffusion assays revealed that Ag NPs exhibit bactericidal activity against *Klebsiella pneumoniae* and *Escherichia coli* strains. The authors concluded that aqueous leaf extracts of *O. majorana* and *S. officinalis* support Ag NP fabrication, with great antibacterial efficiency against MDR bacterial pathogens [15,16]. Also, Allend and collaborators reported the antibacterial activity of Ag NPs based on *Fusarium oxysporum*, alone or in combination with colistin against carbapenem-resistant AB strains [17]. More recently, Camargo and collaborators conducted a study to develop a compound by functionalizing Ag NPs with amikacin against MDR AB strains. The inhibitory activity of the obtained formulation was assessed by performing susceptibility tests using amikacin-resistant AB strains. Susceptibility testing revealed a great potential in inhibiting AB strains [18]. In a series of experimental assays, Ag NPs have exhibited potent bactericidal properties against various pathogenic strains, including *Escherichia coli*, *Pseudomonas aeruginosa*, and *A. baumannii*. These two separate research teams have independently confirmed these findings, suggesting that Ag NPs hold significant promise as a potential stand-alone therapy or as a complementary approach alongside antibiotics and antifungal agents in the fight against bacterial infections [19,20]. Other current studies reported the antibacterial efficiency of Ag NPs synthesized using the flavonoid extract of *Perilla frutescens*, *Calvatia gigantea*, *Mycena leaiana*, and *Ocimum sanctum*. These formulations showed significant inhibition of several pathogens, including *Listeria monocytogens*, *Enterococcus faecalis*, *A. baumannii*, *Staphylococcus aureus*, *P. aeruginosa*, *K. pneumoniae*, *Proteus mirabilis*, *Enterobacter cloacae*, and *E. coli* [21,22,23]. This research underscores the potential utility of Ag NPs in antimicrobial applications, particularly in addressing MDR strains. However, it is critical to note that while these results are promising, further studies, including clinical trials, are required to assess their safety and efficacy in practical medical settings.

In this context, this study aims to demonstrate the effectiveness of Ag NPs as therapeutic alternatives against a significant number of MDR AB isolated for four consecutive years from intra-hospital infections (IHIs) and different aquatic environments.

## 2. Materials and Methods

### 2.1. Bacterial Strains

**Figure 2:**
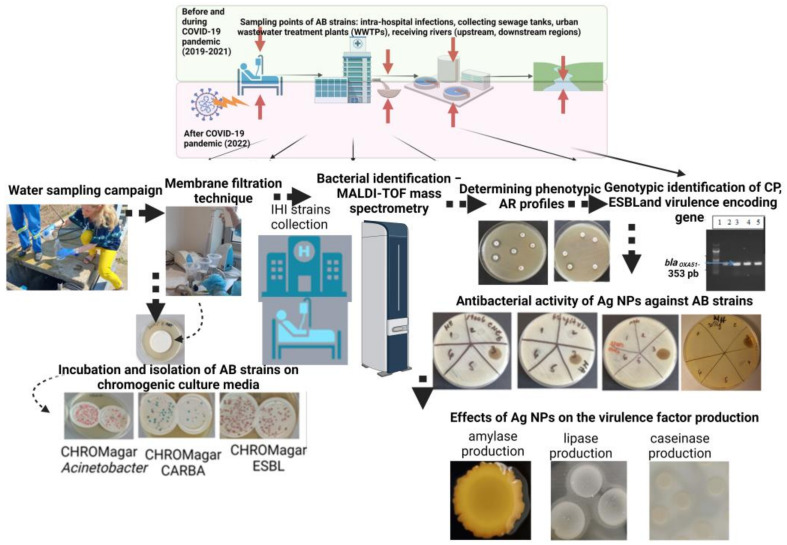
Graphic representation of the workflow (created with Biorender.com; accessed on 18 September 2023).

In total, 132 AB strains were selected from a collection of 568 wastewater (WW) and surface water (SW) samples between March 2019 and August 2022 from three representative Romanian regions (north-eastern (N-E), central and western (C-W), and southern (S)) (Table S1, see Supplementary Material). The strains were isolated on chromogenic media (CHROMagar Acinetobacter) or chromogenic media supplemented with cephalosporins (CHROMagar ESBL) and carbapenems (CHROMagar CARBA) (Rambach, France) using a membrane filtration technique, as previously described [24]. The colonies (total number and the resistant ones) developed after cultivation at 37 °C for 24 h in aerobic conditions were subsequently inoculated on the corresponding media for confirmation: total *Acinetobacter* microbial load (CHROMagar Acinetobacter), extended-spectrum β-lactamase (ESBL) (CHROMagar ESBL), or carbapenemase (CP)-producing phenotypes (CHROMagar CARBA). All strains were identified with MALDI-TOF-MS (Bruker system). In the same time frame as the collection of the water samples, AB clinical strains were isolated from intra-hospital infections (IHIs) that occurred in the units discharging the wastewater in the sampled WWTPs (Figure 2). All AB strains used in this study were maintained in a Revco LegaciTM Refrigeration System (Copeland, UK) at −80 °C on Muller Hinton medium supplemented with 20% glycerol. Before their use, AB strains were cultivated for 24 h at 37 °C on Plate Count Agar (PCA) medium.

### 2.2. Antibiotic Susceptibility Profiles

The antibiotic susceptibility profiles of 132 AB strains were determined using the standard disc diffusion method according to Clinical and Laboratory Standards Institute (CLSI) guidelines for 2022 and tested the following antibiotics: ampicillin-sulbactam (SAM, 20 μg); cefepime (FEP, 30 μg); minocycline (MH, 30 μg); aztreonam (ATM, 30 μg); meropenem (MEM, 10 μg); imipenem (IMP, 10 μg); doripenem (DOR, 10 μg); ciprofloxacin (CIP, 5 μg); amikacin (AK, 30 μg); ceftazidime (CAZ, 30 μg); and gentamicin (CN, 10 μg) [25]. The strains were further classified according to their MDR profile, considering the classes of antibiotics proposed by Magiorakos et al. [26]. The tested antibiotics were grouped into 7 classes: aminoglycosides (AK and CN), antipseudomonal carbapenems (IMP, MEM, and DOR), antipseudomonal fluoroquinolones (CIP), extended-spectrum cephalosporins (CAZ and FEP), penicillins + β-lactamase inhibitors (SAM), and tetracyclines (MH). However, we categorized the strains according to the following criteria: (i) non-MDR phenotype (susceptible to all or resistant to 1–2 classes of antibiotics listed above), (ii) MDR to 3–4 classes, and (iii) MDR to 5–7 classes of antibiotics.

### 2.3. Biofilm Formation Assay

To assess the biofilm formation capacity, 10% of 0.5 McFarland suspension from AB cultures grown for 24 h at 37 °C on PCA solid media was inoculated in Tryptic Soy Broth (TSB) culture media supplemented with 2.5% glucose, final volume 100 μL in 96-well microtiter plates, followed by incubation for 24 h at 37 °C [27]. The plates were then washed three times with phosphate-buffered saline (PBS). In order to facilitate the fixation, cells were treated with methanol for 5 min. Then, staining with crystal violet (1%) solution was performed for 15 min. Finally, after three washing steps with PBS, cells were re-suspended with 33% acetic acid solution. Absorbance (OD) values were determined using a spectrophotometer at 490 nm. All samples were analyzed in triplicate.

The relative biofilm-forming capacity of each strain was assessed according to Stepanović et al. [27]. Namely, a cut-off OD value (ODc) was set at three standard deviations over the mean of the negative controls. Then, the mean OD value for each set of triplicates was calculated, along with the standard deviation. Strains were categorized as follows: non-biofilm producers [OD ≤ ODc], weak biofilm producers [ODc < OD ≤ (2 × ODc)], moderate biofilm producers [(2 × ODc) < OD ≤ (4 × ODc)], and strong biofilm producers [OD > (4 × ODc)].

### 2.4. Characterization of Resistance and Virulence Profiles of AB Strains

Simplex and multiplex PCR were used to screen the presence of carbapenem- and cephalosporin-encoding genes (*bla*_VIM_, *bla*_IMP_, *bla*_NDM_, *bla*_KPC_, *bla*_GES_, *bla*_SHV_, *bla*_TEM_, *bla*_CTX-M_, *bla*_PER_, *bla*_VEB_, *bla*_OXA-23_, *bla*_OXA-24_, *bla*_OXA-58_, *bla*_OXA-235_, and *bla*_OXA-51_), as well as for VM detection (*ompA*, *epsA*, *csuE*, *bap*, and *bfmS*) using a DNA template extracted with an alkaline extraction method, specific primers, and amplification programs (Table 1) and checked with gel electrophoresis [28]. Positive strains for the investigated genes were used as the positive controls.

### 2.5. Synthesis of Ag NPs

Three types of Ag NPs were synthesized using classical, Turkevich, and solvothermal methods [29]. Regarding the synthesis of Ag NPs using the hydrothermal method (Ag NPsol), 0.888 g of PVP K30 was added to 80 mL of PEG 400, stirred, and heated to 80 °C until the obtained solution became transparent. When the temperature reached 80 °C, quickly, 2 mL of AgNO_3_ with a concentration of 0.5 M was added to the mixture, continuing the stirring. The appearance of a dark yellow coloration was observed, and the mixing was continued at 80 °C until the appearance of a dark brown coloration. The mixture was poured into a Teflon vat, and the temperature was raised to 220 °C while maintaining a constant pressure of 1 bar for 2 h. The obtained mixture, reddish brown in color, cooled down and was then removed from the vat. The concentration of the obtained NPs was 1 mg/mL.

### 2.6. Ag NP Characterization Techniques

Scanning electron microscopy (SEM) images were recorded using a Quanta Inspect F FEI (Lausanne, Switzerland) microscope equipped with an EDS spectrometer, with the samples covered with a thin film of silver; high-resolution transmission electron microscope (TEM) images were recorded using a TecnaiTM G2 F30 S-TWIN HRTEM from FEI equipped with EDS and SAED. The microscope was operated in transmission mode at 300 kV with TEM point resolution of 2 Å and line resolution of 1 Å. TEM and SEM were used to determine the morphology of the materials (surface appearance, especially particle size and shape).

### 2.7. Qualitative Screening of the Antibacterial Activity of the Ag NPs

An adapted diffusion method using 10 μL of Ag NPs (Ag NPc = 0.01 mg/mL; Ag NPt = 0.1 mg/mL; and Ag Npsol = 1 mg/mL) was performed on Mueller Hinton (MH) for a total of 132 AB strains (0.5 McFarland inoculum from cultures of 24 h at 37 °C on PCA solid media) selected from a collection of 568 AB strains previously isolated from IHIs, SW samples, and WW samples from three different geographic locations in Romania. After 24 h at 37 °C, the growth inhibition diameters were measured and converted into arbitrary units, as previously described [30]. A *P. aeruginosa* ATCC 27853 strain purchased from the American Type Culture Collection (ATCC, Manassas, VA, USA) and meropenem (64 μg/mL) were used as a positive control, according to CLSI 2022 guidelines [25].

### 2.8. Quantitative Evaluation of the Antibacterial Activity of the Ag NPsol

The antimicrobial activity was measured in MH Broth medium using serial two-fold microdilutions of the Ag NPsol in 100 μL of broth medium seeded with 0.5 McFarland inoculum (from cultures of 24 h at 37 °C on PCA media) and determining the minimum inhibitory concentration (MIC) values after the incubation at 37 °C for 24 h. Untreated AB cultures were used as a positive control of growth and *P. aeruginosa* ATCC 27853 was used to validate the obtained results, cultivated in the presence of meropenem, antibiotic serial diluted starting from 64 μg/mL stock solution.

### 2.9. Effect of Ag NPsol on the Adherence Capacity and Soluble Virulence Factor Production

Microbial strains grown treated with a sub-inhibitory concentration of Ag NPsol (MIC/2) were evaluated for their capacity to adhere and to secrete soluble virulence factors such as hemolysins, lecithinase, amylase, lipase, caseinase, gelatinase, and esculin hydrolysis, using 10 μL of 0.5 McFarland suspension from AB strains grown for 24 h at 37 °C on PCA media (untreated culture, serving as growth control and treated with Ag NPs) and incubated for 24 h at 37 °C, as previously described [31,32].

The influence of Ag NPsol on hemolysins, lecithinase, amylase, lipase, caseinase, gelatinase, and esculin hydrolysis was evaluated by using the following relation:

(1) $$\mathrm{Inhibition}\;(\%)=\frac{D2-C2}{D1-C1}\times 100,$$

where:

C1—colony diameter of strain control;
D1—clear/brown/white/yellow precipitate/ring surrounding the culture spot/yellow zone diameter of strain control;
C2—colony diameter of sample;
D2—clear/brown/precipitation/yellow zone diameter of sample.

For the adherence capacity inhibition assay, the protocol was similar to the biofilm formation assays (Section 2.3), with the mention that AB strains were cultivated in the presence of sub-inhibitory concentrations of Ag NPsol.

The adherence inhibition percentage was calculated as follows:

Adherence inhibition % = (As − Ablank/Ac − Ablank) × 100(2)

where:

As = the absorbance of the microbial adherence of AB strains treated with Ag NPsol;
Ac = the absorbance of the microbial adherence of untreated AB strains.

### 2.10. Extracellular Nitric Oxide Quantification

Nitric oxide content was determined using a spectrophotometric assay which was described by Quinteros et al. [33] with minor changes. The AB strains were grown for 24 h at 37 °C in Muller Hinton broth containing a sub-inhibitory concentration of Ag NPsol (MIC/2). We used culture media control, strain control, and sample control without microbial strains. For the quantification of nitric oxide, a calibration curve was performed with NaNO_2_ (0–100 μM R2 = 0.9995).

### 2.11. Determination of the Interaction between the Ag NPs and Conventional Antibiotics

A disc diffusion method using 0.5 McFarland inoculum obtained from AB strains grown for 24 h at 37 °C on PCA media, conventional antibiotics recommended by CLSI 2022 guidelines, and antibiotics supplemented with Ag NPsol (5 μL; 1 mg/mL) were applied in order to determine the inhibition zone diameters (IZDs) after 24 h of incubation at 37 °C. The obtained results were expressed as percentage change in IZD compared to controls (IZD for antibiotics) and recorded as synergistic effect for IZD ≥ 19%, additivity for IZD between 0 and 19%, and antagonism for IZD < 0, as previously described [34].

### 2.12. Statistical Analysis

Data obtained in triplicate/duplicate were expressed as means ± SD. Statistical analysis was performed using GraphPad Prism v10. Data were analyzed using the two-way ANOVA for extracellular nitric oxide content, adherence, and virulence factor inhibition. The correction of multiple comparisons for nitric oxide content was carried out using Tukey’s method with a single pooled variance, the comparison being sample vs. strain control or sample control. The correlations between microbial adherence and the extracellular nitric oxide content and the adherence and NO consumption were achieved through the Pearson correlation. Tukey’s method was used for MIC value evaluation, the comparison being performed between the mean MIC values for the isolation sources of each region. For the overall assessment of the sub-inhibitory concentration of Ag NPsol inhibition effect on AB adherence to inert substratum, the correction method chosen was Dunnett’s multiple comparisons test, with the comparison being performed between control values vs. MIC/2 and MIC/4 values obtained for each isolation source. At the individual level, the correction of multiple comparisons for determining the effect of Ag NPsol on the adherence capacity and soluble virulence factor production was carried out using the Dunn–Šidák method. The level of statistical significance was set at *p* < 0.05.

## 3. Results

### 3.1. Characterization of Ag NPs

Three types of Ag NPs synthesized using classical, Turkevich, and solvothermal methods were characterized with FTIR, SEM, TEM, DLS, and XRD methods [29].

**Figure 3:**
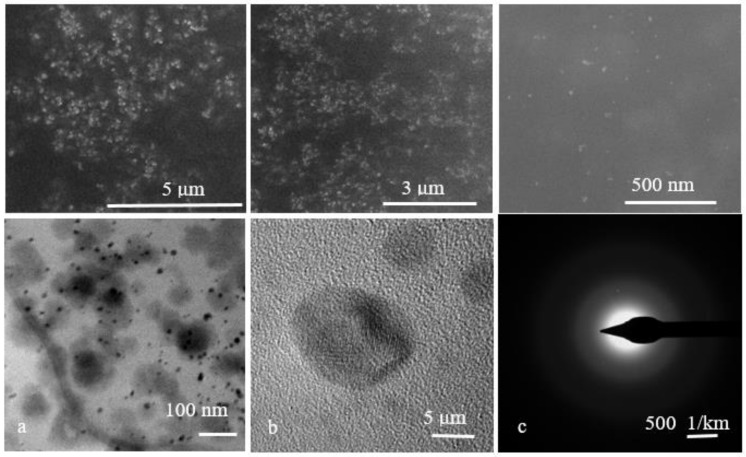
Representative SEM images recorded at different magnifications (**top**) and TEM (**bottom** (**a**)), HRTEM (**bottom** (**b**)), and SAED (**bottom** (**c**)) images of the Ag NPs.

SEM images of the recorded Ag NPs showed small particles, mostly spherical in shape and with a size in the nanometric range (Figure 3 top) [35,36].

The TEM images of the Ag NPs confirmed at a lower range the information from SEM, meaning the spherical shape, the polydispersed nature of these nanoparticles, and the agglomeration-free formation from the solution. The size was also determined, with the Ag NPs being found to be in the range of 10–20 nm. The selected area electron diffraction (SAED) pattern (Figure 3 bottom) showed polycrystalline diffraction rings, which indicate the highly crystalline nature of Ag NPs [35].

### 3.2. Phenotypic Antimicrobial Resistance (AR) Profiles

#### 3.2.1. North-East Region

**Figure 4:**
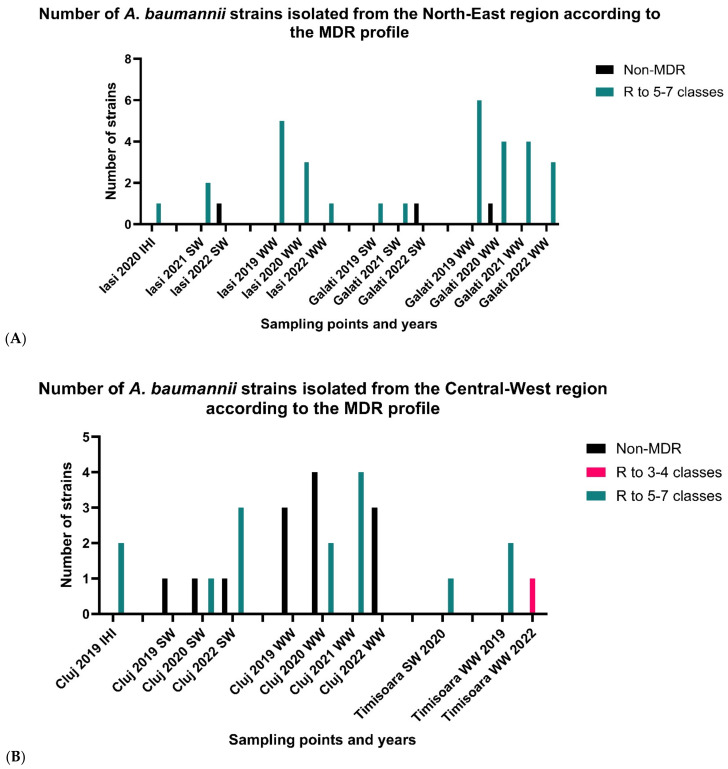

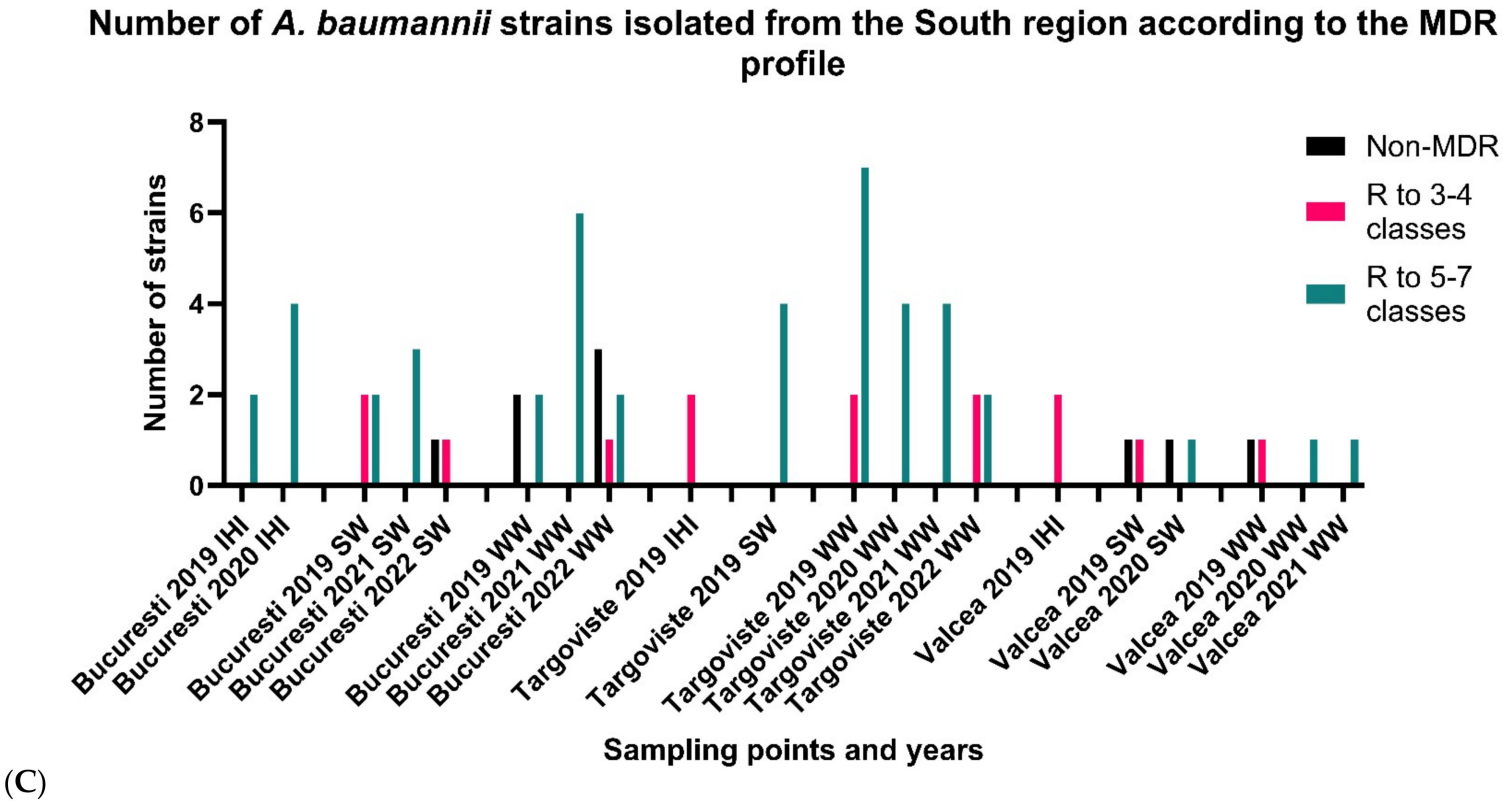
Number of *A. baumannii* strains isolated from IHIs, SW samples, and WW samples in the north-east (**A**), central-west (**B**), and southern (**C**) regions of the country, categorized according to their MDR profile: non-MDR (susceptible to all or resistant to 1–2 classes of antibiotics), MDR to 3–4 classes of antibiotics, and MDR to 5–7 classes of antibiotics, according to [26].

The comparative study of the AR profiles according to the isolation sources and period of isolation demonstrated the following for a total of 35 AB strains recovered from the N-E region by location: for Iași, the maximum resistance level corresponded to AB strains isolated in 2021, 2020, and 2019 from all investigated sources for all tested antibiotics with the exception of FEP and CAZ (93.75%), IMP (80%), and CN (75%). Regarding the last sampled year (2022), a decreasing level for all tested antibiotics was noticed. In the second investigated location—Galați—AB strains recovered in 2021 from WW samples and SW samples revealed the PDR phenotype. The opposite was found for the strains isolated in 2022 from the same isolation sources, which showed the lowest resistance levels (i.e., MH (25%) and FEP (12.5%)). In total, 89% of the strains isolated from the north-eastern region of the country were grouped as MDR to 5–7 classes of antibiotics and 11% of the strains were grouped as non-MDR, while there were no strains in the MDR to 3–4 classes category (Figure 4A). It was noticed that there were a decreasing number of strains isolated across the sampling period. The most common pattern included susceptibility to SAM, MH, and cephalosporins.

#### 3.2.2. Central-Western Regions

The comparative analysis of the AR profiles according to the isolation sources and period of isolation demonstrated the following for a total of 29 AB strains recovered from C-W Romania by location: for Cluj, in isolated from IHIs in 2019 > AB strains decreasing order of the resistance, there were AB strains isolated from WW samples revealed in 2021 (PDR phenotype) > AB strains recovered in 2019 and 2020 from SW samples > AB strains recovered in 2022 from WW samples. For Timișoara, the highest resistance levels to carbapenems (MEM and IMP) corresponded to AB strains isolated in 2019 and 2020, respectively. Out of a total of 29 strains, 52% were included in the MDR to 5–7 classes of antibiotics category, 3% of the strains were included in the MDR to 3–4 classes of antibiotics category, and 45% were included in the non-MDR category. Similarly to the strains isolated from the north-eastern region, the most common pattern noticed included susceptibility to SAM, MH, and cephalosporins while being resistant to other classes of tested antibiotics. Although the low number of the strains does not allow us to draw certain conclusions, an increasing trend in the number of resistant strains in the sampled points was noticed (Figure 4B).

#### 3.2.3. South Romania

The comparative analysis of the AR profiles according to the isolation sources and year demonstrated the following for a total of 68 AB strains recovered from south Romania by location: in Bucharest, the top resistance levels were shown by AB strains isolated from IHIs in 2019 and 2020 and those recovered from WW samples in 2021 (all being PDR), and the lowest resistance levels corresponded to AB strains isolated from SW samples in 2022. For Târgoviste, in decreasing order of resistance, were the AB strains isolated from WW samples recovered in 2021 > AB strains isolated from WW samples recovered in 2020 > AB strains recovered in 2019 from all sampling points > AB strains recovered from WW samples in 2022 (Figure 4C). AB strains isolated from WW samples from Râmnicu Vâlcea in 2020 and 2021 exhibited the XDR phenotype. Out of 68 strains from south Romania, 66% were grouped in the MDR to 5–7 classes of antibiotics category, 21% of the strains were grouped in the MDR to 3–4 antibiotics category, and the remaining 13% were classified as non-MDR (Figure 4C). Across the period of sampling, a decreasing tendency in the isolation of highly resistant strains was noticed. The most common pattern included susceptibility to SAM and MH while being resistant to antibiotics in other tested classes.

### 3.3. Genotypic AR Profiles

A total of 44 AB strains with the MDR phenotype (*n* = 35; 79.54%) and non-MDR strains (*n* = 9; 20.45%) isolated from different Romanian regions and isolation sources and exhibiting different acquired β-lactamases: OXA23, OXA-24 (IHI/WW AB from S); OXA-23 (SW AB from S/C-W and WW AB from S/N-E/C-W); OXA-24 (IHI AB from S; WW AB from S/N-E/C-W); OXA-24, TEM (SW AB from S/N-E and WW AB from N-E); OXA-24, PER (SW AB from N-E); and TEM (SW AB from N-E) were associated with the presence of VMs involved in the biofilm formation of AB, such as the outer membrane protein A (OmpA), biofilm-associated protein (bap), chaperon-usher pilus (csu), or regulation systems (bfmS) (Figures S2–S11, see Supplementary Material).

Regarding the biofilm production capacity by isolation source, AB strains belonged to the following categories: AB strains from IHIs have a moderate ability to adhere regardless of the susceptibility pattern. In the case of AB strains from SW samples, the adherence capacity varies from weak to moderate, and even non-adherent strains were identified; furthermore, non-MDR AB strains isolated from SW samples demonstrate a moderate to strong adherence capacity, which confirms that, usually, the strains can express either resistance or VMs. The adherence patterns of MDR AB strains isolated from WW samples have a higher degree of variability, with some isolates being described as strong biofilm-producing strains; thereby, the adherence capacity is a characteristic which depends on the environmental conditions to which the strains were exposed, proving that the selective pressure of the isolation source has a determining character. AB strains from IHI, WW, and SW samples were positive for biofilm-related genes, such as *ompA*, *bfmS* (93.18% each), *csuE* (90.90%), and *bap* (43.18%). The highest number of biofilm-related genes were present in 38.63% of MDR AB, weak, and moderate biofilm producers (*ompA-csuE-bfmS-bap*) (Table 2).

### 3.4. Antimicrobial Activity of Ag NPs

**Figure 5:**
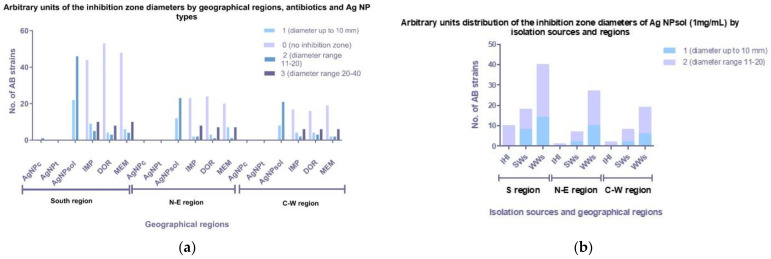
Graphic representation of the distribution of inhibition zone diameters in arbitrary units by geographical region (**a**) and isolation source (**b**).

The qualitative screening of the antimicrobial activity of Ag NPs showed that Ag NPsol inhibited the growth of all tested AB strains with different efficiency (69% of AB strains for arbitrary unit identification 2 and 31% of the strains for arbitrary unit identification 1) (Figure 5a; Figure S1, see Supplementary Material). It was noticed that the majority of AB strains were isolated from WW samples (*n* = 86; 65%) (Figure 5b).

**Figure 6:**
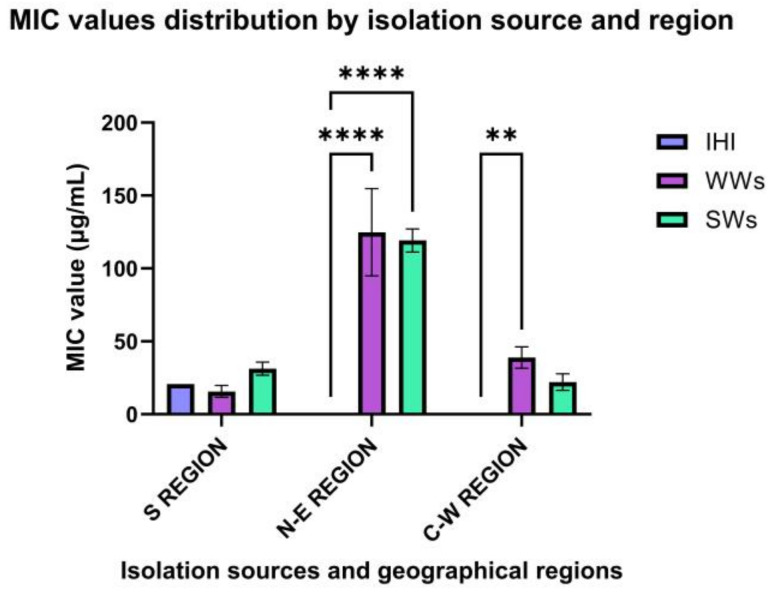
Graphic representation of MIC average values obtained for the AB strains from different isolation sources and geographical regions (** *p* < 0.001, **** *p* < 0.0001) (Tukey’s method).

Further, the quantitative evaluation of the antimicrobial efficiency of Ag NPsol against AB strains by isolation source and year revealed that the highest efficiency corresponded to AB strains isolated from the S and C-W regions (19.41 μg/mL and 31.46 μg/mL, respectively). The most susceptible were AB strains isolated in 2021 and 2020, respectively, from WW (17.57 μg/mL/9.75 μg/mL) and SW samples (15.62 μg/mL/20.83 μg/mL) (Figure 6).

### 3.5. Ag NPsol Effect on Adherence Capacity

**Figure 7:**
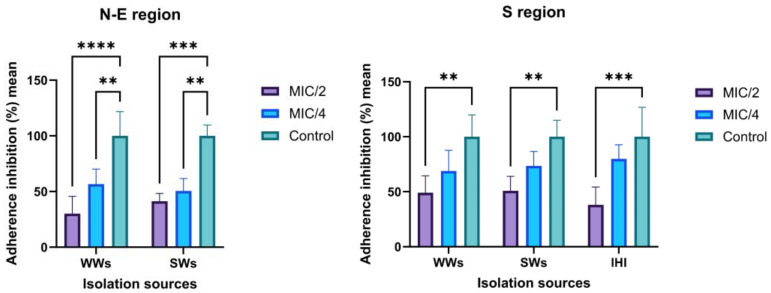
Graphic representation of adherence inhibition percentage mean values obtained for the AB strains from different isolation sources and geographical regions cultivated in the presence of MIC/2 and MIC/4 Ag NPs (** *p* < 0.001, *** *p* < 0.001, **** *p* < 0.0001) (Dunnett’s multiple comparisons test).

AB isolated from the S and N-E regions showed high potential for biofilm development. Ag NPsol at sub-inhibitory concentrations (minimum inhibitory concentration, MIC/2, and MIC/4) inhibit AB adherence to inert substratum. The best results were obtained for AB strains isolated from WW and SW samples from the N-E region at MIC/2 value (34.78%/39.43%) and IHIs from the S region (Figure 7). According to the obtained results, the exposure of the isolates to the MIC/2 value induces a significant change in the adhesion capacity to the inert substrate both for the strains from the southern region and those from the north-eastern region. For further analysis at an individual level, MIC/2 concentration was chosen as the optimal maximum value that does not disturb the growth of the strains but might interfere with their ability to adhere or secrete soluble virulence factors.

**Figure 8:**
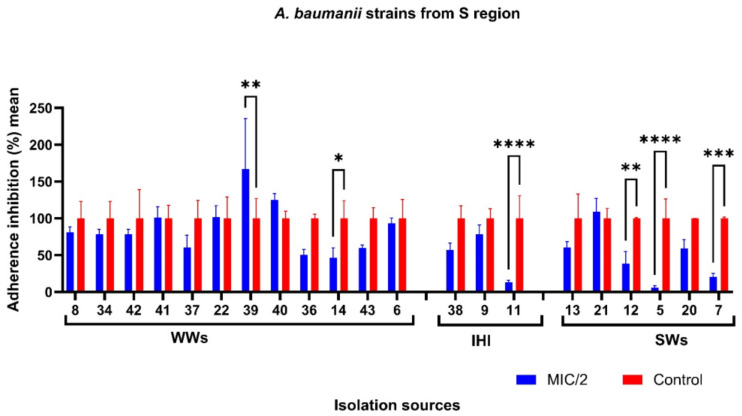
Adherence inhibition percentage values for Ag NPsol compared to the untreated isolates from the S region (* *p* < 0.05, ** *p* < 0.001, *** *p* < 0.001, **** *p* < 0.0001) (Dunn–Šidák method).

With the exception of six non-biofilm-producing AB strains, all isolates were investigated regarding the influence of Ag NPsol against the adherence capacity. According to Figure 8, the adherence capacity of five AB strains isolated from IHI, SW, and WW samples in southern Romania was significantly inhibited in the presence of sub-inhibitory concentrations of Ag NPsol. It was noticed that for the AB strain encoded 39 the adherence capacity was stimulated (adherence inhibition percentage = 167.25).

**Figure 9:**
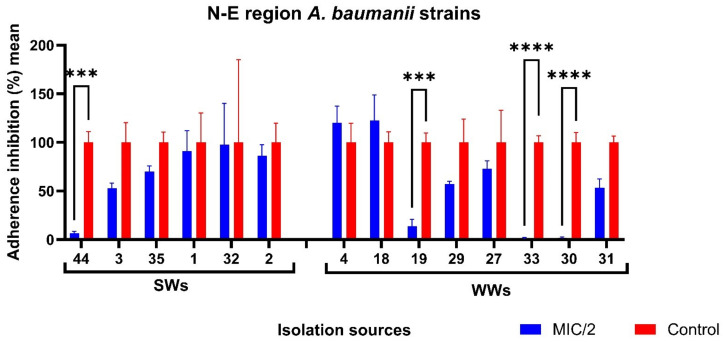
Adherence inhibition percentage values for Ag NPsol compared to the untreated isolates from the N-E region (*** *p* < 0.001, **** *p* < 0.0001) (Dunn–Šidák method).

For N-E Romania, the adherence capacity of three AB strains isolated from WW samples (adherence inhibition percentage = 0.23–13.85) and one strain from SW samples (adherence inhibition percentage = 6.56) was significantly inhibited in the presence of sub-inhibitory concentrations of Ag NPsol (Figure 9).

In the case of AB strains from central-western Romania, no inhibition effect was observed for any of the AB strains (*n* = 5).

### 3.6. The Modulation of Virulence Factor Capacity by Ag NPsol

From the tested AB strains, 93.18% produced caseinases, 50% lipases, 95.45% lecithinase, and 77.27% amylase. None of the tested AB strains are able to produce hemolysins or gelatinases or to hydrolyze the esculin (Table 3).

Starting with these results, strains producing caseinase and lipase were further selected to assess the influence of the Ag NPsol on the production of each of them. Regarding caseinases, the enzymatic production was significantly inhibited by the Ag NPsol in the cases of three AB strains isolated from WW samples from the N-E region (*p* < 0.0001) and S region (*p* < 0.05) and SW samples from the N-E region (*p* < 0.0001).

Lipase production was completely inhibited in the case of two isolates from the N-E region (*p* < 0.001). However, the presence of sub-inhibitory concentrations of Ag NPsol did not significantly influence the ability of the other strains to secrete these enzymes. Thus, the influence of the solution on the strains’ ability to secrete soluble virulence factors in the culture medium varies depending on the metabolic characteristics of the tested isolates. In the case of amylase and lecithinase, the hydrolyze zone diameter was equal to the culture spot; therefore, the inhibitory effect could not be quantified.

### 3.7. Correlation between NO Release and Antibiofilm Effect of Ag NPs

**Figure 10:**
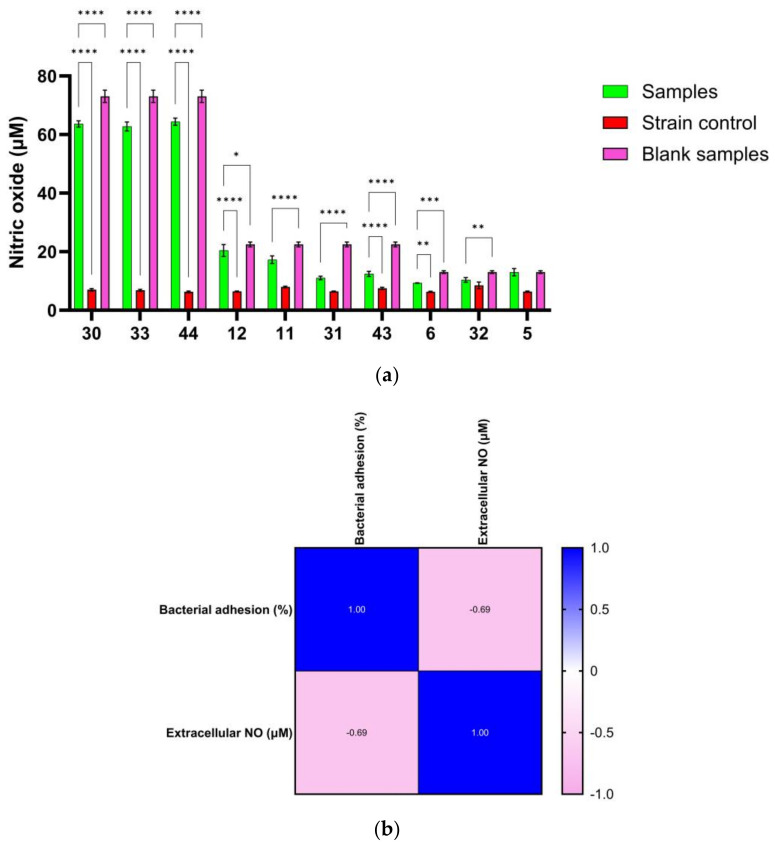
Extracellular NO content determined by Griess’s reaction for Ag NPs in presence of *A. baumanii* strains (**a**) (Tukey’s method, * *p* < 0.05, ** *p* < 0.01, *** *p* < 0.001, **** *p* < 0.0001); and Pearson correlation between extracellular NO and anti-adherence activity (**b**).

RNI can bind to various targets, including lipids, metal centers, protein tyrosine residues, nucleotide bases, and thiols. NO immediately affects enzymes that interfere with bacterial respiration, growth arrest, and DNA synthesis suppression. To deal with environmental changes, bacteria have specialized and all-purpose defense mechanisms, such as stressor detoxification and defense and repair systems [37]. Since various antibiotics work by oxidative mechanisms that cause bacterial mortality by oxidizing macromolecules such as lipids, DNA, and proteins [38], nanoparticles may have a lethal effect on bacterial structures. In Figure 10a, a significant increase in the extracellular NO content compared to the strain control for variants 30, 33, 34, 43, and 12 (*p* < 0.0001) and 6 (*p* < 0.001) can be seen. The significant decrease in extracellular NO compared to the test control without the strain proved significant for variants 30, 33, 44, 11, 31, 43 (*p* < 0.0001), 12 (*p* < 0.05), 6 (*p* < 0.001), and 32 (*p* < 0.01), which may suggest that bacteria used a multitude of enzymes and complex mechanisms to sense, capture, and transform NO into less reactive compounds. Due to its high reactivity, NO exists only at deficient cell concentrations and is rapidly transformed by denitrification and aerobic oxidation [39].

Although studying bactericidal action in planktonic bacteria is a promising method for evaluating new antibacterial drugs, the eradication of microbial biofilms is required to assess their therapeutic value. The antibiofilm activity of exogenous NO released by NO donors (e.g., Ag NPs) with low molecular weight is dose-dependent, with eradication concentrations also dependent on bacterial species and potential genetic alterations. NO causes biofilm development and can disperse established biofilms through cell signaling pathways at low levels (10^−12^–10^−9^ M). High NO concentrations (10^−6^–10^−3^ M) remove biofilms by directly killing embedded bacteria [40]. The link between the extracellular concentration of NO released at MIC/2 levels and microbial adherence revealed that the higher the extracellular concentration of NO, the stronger the inhibition of microbial adherence. The Pearson correlation revealed that one variable grows as the other declines (*r* < 0). The resulting *p*-value of 0.028 (*p* < 0.05) indicates that the association is not due to random sampling (Figure 10b).

**Figure 11:**
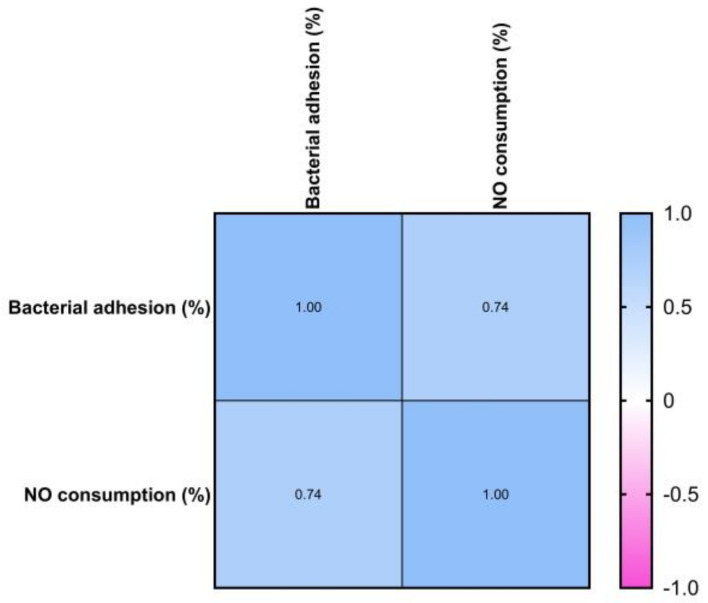
Pearson correlation between NO consumption and anti-adherence activity.

Moreover, by evaluating the NO content in the medium with Ag NPs (blank sample) and the value obtained after incubation with the microorganism in Figure 10a, it can be concluded that part of the NO generated by Ag NPs is consumed. The percentages of consumed NO can be found in Table 4, and the Pearson correlation (Figure 11) shows that the two variables tend to increase or decrease together. Thus, the lower the microbial adhesion, the lower the bacteria’s NO consumption, and practically, the NO content in the extracellular environment is higher. The bacterial cells of *A. baumanii* metabolize a maximum of 10 μM of nitric oxide released, expressed as NaNO_2_ equivalents.

### 3.8. The Interaction between the Ag NPsol and Conventional Antibiotics

**Figure 12:**
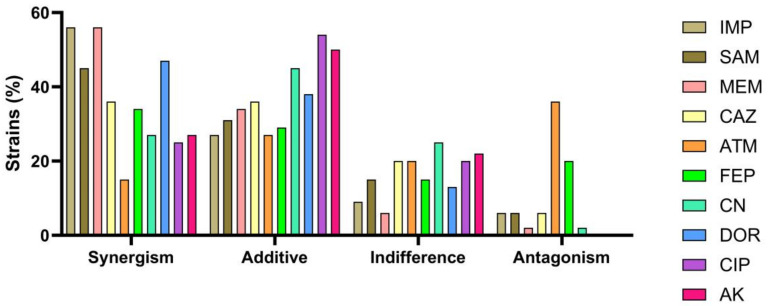
The interaction between antibiotics and Ag NPsol against AB strains isolated from IHIs, WW samples, or SW samples, using the following antibiotics: IMP, 10 μg; SAM, 20 μg; MEM, 10 μg; ATM, 30 μg; CAZ, 30 μg; FEP, 30 μg; CN, 10 μg; DOR, 10 μg; CIP, 5 μg; and AK, 30 μg.

The diameters of the inhibition zone presented by conventional antibiotics against isolated AB strains compared to their combination with Ag NPsol led to a variable inhibition of all the isolates used in this study (Figure S12, see Supplementary Material). Out of the 440 different combinations, 164 (37.27%) were synergistic, 165 (37.5%) were additive, 75 (17.05%) were indifferent, and 36 (8.18%) were antagonistic. Overall, this means that only 8.18% of all combinations were antagonistic, the majority of variants being with the antibiotic ATM (16), followed by FEP (9). Figure 12 shows the percentage of bacteria that proved sensitive to combinations between antibiotics and Ag NPsol, divided according to interaction type: synergistic, additive, indifferent, or antagonistic. The most pronounced synergistic action was for the combinations of Ag NPsol with the following antibiotics: IMP, MEM, DOR, SAM, CAZ, and FEP. In the case of ATM, it can be observed that the combination with Ag NPsol is antagonistic in most cases, and the additive interaction was observed especially for the Ag NP-AK, Ag NP-CIP, and Ag NP-CN variants. The detailed results of the analysis of Ag NPsol behavior in the presence of different antibiotics are shown in Table S2 (see Supplementary Material).

To highlight a possible behavior of the interaction between antibiotics and Ag NPs, a reference strain (*P. aeruginosa* ATCC 27853) was used for which it was observed that no antibiotic–Ag NP combination is synergistic; in fact, 50% of the combinations proved antagonistic, 30% indifferent, and 20% additive (Table S2, see Supplementary Material).

## 4. Discussion

Our obtained results on a significant number of clinical, wastewater, and surface water strains have shown the decrease in MDR level in AB strains isolated from WW and SW samples in 2022 from different Romanian regions compared to previous years.

An important factor in the persistence of infections caused by AB is represented by the biofilm formation capacity [8]. Regarding the adherence capacity of clinical and aquatic Romanian AB strains, our obtained results demonstrated a higher degree of variability, identified as moderate (38.63%), weak (36.36%), and strong (11.36%) biofilm-producing strains. The obtained results highlight that the strong biofilm-producing AB strains showed both MDR and non-MDR phenotypes and were positive for the *ompA* gene responsible for adherence to the host cells, biofilm formation, and AR [41]. According to previous studies, our investigated AB strains from clinical and different aquatic compartments were positive for biofilm-related genes, such as *ompA*, *bfmS* (93.18% each), *csuE* (90.90%), and *bap* (43.18%), responsible for biofilm formation and AR [42,43]. A very high frequency of *ompA* and *bfmS* genes was encountered in our study, with similar results to those reported by Zeighami et al. in 2019 [8] and Thummeepak et al. in 2016 [41].

Regarding the distribution of biofilm-related genes by weak and moderate biofilm-producing MDR AB strains in 38.63%, the presence of four biofilm-related genes (*ompA*-*csuE*-*bfmS*-*bap*) was noticed. In two aquatic AB strains isolated from SW and WW samples from southern and N-E Romania, respectively, it was found that the presence of the ESBL-encoding gene *bla*_TEM_ enhanced biofilm development. Furthermore, in one MDR AB strain isolated from SW samples in N-E Romania, it was shown that biofilm formation was enhanced by the ESBL-encoding gene *bla*_PER_, as previously demonstrated [41], and also by the acquired CP OXA-24.

In recent years, interest has increased in applying NPs as therapeutic regimens. In particular, Ag NPs have attracted much attention in the scientific field. Silver has always been used against various diseases; in the past, it was used as an antiseptic and antimicrobial against Gram-positive and Gram-negative bacteria due to its low cytotoxicity. The antimicrobial activity of Ag NPs demonstrated that the highest efficiency of Ag NPsol corresponded to AB strains isolated from southern and central Romania, the most susceptible being AB isolated in 2021 and 2020 from WW and SW samples. Furthermore, Ag NPsol in sub-inhibitory concentrations inhibited the adherence capacity of AB strains (*n* = 5; 11% and *n* = 4; 9%, respectively) isolated from all isolation sources/WW and SW samples in S/N-E Romania. Our obtained results demonstrated that Ag NPsol inhibited the caseinase and lipase production in 3/2 AB strains isolated from WW/SW samples from N-E and S/N-E regions, respectively.

Consistent with our results, previous studies have shown the efficiency of Ag NPs in inhibiting biofilms produced by different microorganisms and eradicating MDR AB strains. Hetta and collaborators conducted a study to assess Ag NPs’ impact on bacterial growth, virulence, and biofilm-related gene expression in *A. baumannii* strains isolated from wound infections. Their results showed silver’s efficiency in inhibiting the strong biofilm-producing AB strains. Also, overnight incubation of silver in well-formed biofilms led to their partial removal, concluding that Ag NPs could be used as a biofilm-disrupting agent [10]. Similarly, Rezania et al. [44] investigated the effect of silver and gold NPs on the expression of *bap* and *csu* genes responsible for biofilm formation. They observed that Ag NPs have an inhibitory effect against AB biofilm formation and modulate the gene expression. These results highlight the potential of Ag NPs in preventing biofilm formation and treating infections with or without antibiotics [44]. Singh and collaborators investigated the antibacterial activity of Ag NPs against *A. baumannii* planktonic bacteria embedded in biofilms. They observed a synergistic interaction of Ag NPs with doxycycline, tetracycline, and erythromycin. NPs exhibited significant biofilm disruption activity. These NPs affected bacterial growth and distorted cellular morphology. Intracellular oxidative stress, induced in the presence of Ag NPs, also rendered bacteria susceptible to NPs [11]. Khaled et al. [45] investigated the synergistic action of colistin, imipenem, and Ag NPs against PDR AB strains isolated from IHIs. The combinations of colistin and Ag NPs or imipenem and Ag NPs resulted in synergistic action that reduced the MICs of colistin, imipenem, and Ag NPs. Also, the combinations of colistin and imipenem had high synergistic action [45].

In our obtained results, a synergistic effect (37.27% of the cases) was obtained in the case of Ag NPsol with carbapenem antibiotics (IMP, MEM, and DOR), cephalosporins (CAZ and FEP), and penicillins + β-lactamase inhibitors (SAM). Similarly, two recent studies demonstrated the synergistic action between Ag NPs, imipenem, aminoglycosides, and ceftriaxone. The MIC values of imipenem-conjugated Ag NPs against resistant *A. baumannii* were decreased in a dose-dependent manner and were based on the presence of resistant genes. The results suggest that imipenem–Ag NPs, ceftriaxone–Ag NPs, and aminoglycosides–Ag NPs are potent antibacterial agents against MDR AB [19,46,47].

Barabadi et al. [48] evaluated the antibacterial and antibiofilm activity of *Penicillium chrysogenum*-derived Ag NPs against AB clinical and reference strains. Ag NPs exhibited antibacterial and antibiofilm activity against both categories of investigated bacteria, inhibiting the biofilm formation capacity [48]. Allend and collaborators investigated the antibacterial activity of biogenic Ag NPs (Bio-Ag NPs) alone or in combination with polymyxin B against several carbapenem-resistant AB (CRAB) strains. They observed that Bio-Ag NPs exhibited antibacterial activity, and the combination of polymyxin B and Bio-Ag NPs presented synergy against four of the five strains tested and additivity against one strain in the checkerboard assay. In addition, Bio-Ag NPs demonstrated a bactericidal effect against all CRAB isolates within 1 h [17]. Shah et al. [49] demonstrated the antibacterial activity of Ag NPs against the majority of the investigated CRAB clinical strains, with MIC values very similar to those obtained in this study [49].

Recently, Camargo et al. [18] developed a nano-drug from Ag NPs functionalized with amikacin against MDR AB. Ag NPs were produced using the bottom-up methodology and functionalized with amikacin modified by carbodiimide-based chemistry, forming AgNPs@Amikacin. Susceptibility tests were performed using amikacin-resistant AB strains to assess the bacteriostatic and bactericidal potential of the developed nano-drug. This compound reduced the metabolic activity of AB at rates ≥50%, characterized by minimal biofilm inhibition concentrations. These results demonstrated the promising development of a new nano-drug with lower concentrations, less toxicity, and greater efficacy against MDR AB [18].

Two recent studies aimed to synthesize, characterize, and investigate the antibacterial activity of Ag NPs fabricated using *Camellia sinensis* leaves and extract of *Ocimum sanctum* against MDR AB. These NPs, obtained through green synthesis, exhibited strong antibacterial activity, leading to damage and rupture in bacterial cells [20,21].

## 5. Conclusions

We provide here the first report regarding the potential of Ag NPs to fight MDR AB high-risk carbapenemase (OXA-23 and OXA-24), ESBL producers (TEM and PER), and virulent strains (*ompA*, csuE, bap, and bfmS) circulating between hospital and anthropically polluted water in different Romanian regions. To the best of our knowledge, there are no data regarding the efficiency of Ag NPsol against wastewater or surface water AB planktonic cells or those embedded in biofilms, and our study will contribute to understanding the mechanisms by which Ag NPs interfere with AB spread from clinical to different aquatic environmental compartments and its persistence. In conclusion, Ag NPs can be used as an alternative therapeutic agent, followed by their pharmacokinetics and pharmacognosy.

## Figures and Tables

**Table 1:** The primer sequences, amplicon sizes, and amplification programs of the PCR reaction.

Target Gene	Primer Sequences	Amplicon Size (bp)	Second Amplification Cycle
**Primers used for CP (OXA-23; OXA-24; OXA51; OXA-58; VIM; IMP; NDM; KPC) and ESBL detection (TEM; SHV; CTX-M; VEB; GES; PER)**			
*bla*_OXA-51_	OXA-51F: TAATGCTTTGATCGG CCT TG OXA-51R: TGGATTGCACTTCATCTTGG	353	30× (95°—30 s, 52°—40 s, 72°—50 s)
*bla*_OXA-23_	OXA-23F: GAT CGG ATT GGA GAA CCA GA OXA-23R: ATT TCT GAC CGC ATT TCC AT	501	
*bla*_OXA-24_	OXA-24F: GGT TAG TTG GCC CCC TTA AA OXA-24R: AGT TGA GCG AAA AGG GGA TT	242	
*bla*_OXA58_	OXA-58F:AAGTATTGGGGCTTGTGCTG OXA-58R:CCCCTCTGCGCTCTACATAC	599	
*bla*_OXA-235_	OXA-235F:TTGTTGCCTTTACTTAGTTGC OXA-235R:CAAAATTTTAAGACGGATCG	700	
*bla*_TEM_	TEM-F: ATA AAA TTC TTG AAG AC TEM-R: TTA CCA ATG CTT AAT CA	1080	30× (95°—30 s, 52°—40 s, 72°—70 s)
*bla*_SHV_	SHV-F: TGG TTA TGC GTT ATA TTC GCC SHV-R: GGT TAG CGT TGC CAG TGC T	868	30× (95°—30 s, 56°—40 s, 72°—60 s)
*bla*_CTX-M_	CTX-M-F: TCG TCT CTT CCA GAA TAA GG CTX-M-R: AAG GAG AAC CAG GAA CCA CG	754	30× (95°—30 s, 56°—40 s, 72°—60 s)
*bla*_VIM_	VIM-F: GAT GGT GTT TGG TCG CAT A VIM-R: CGA ATG CGC AGC ACC AG	389	30× (95°—30 s, 55°—40 s, 72°—50 s)
*bla*_IMP_	IMP-F-GGAATAGAGTGGCTTAAYTCTC IMP-R-GGTTTAAYAAAACAACCACC	232	
*bla*_NDM_	NDM-F: GCA GCT TGT CGG CCA TGC GG GC NDM-R: GGT CGC GAA GCT GAG CAC CGC AT	621	30× (95°—30 s, 55°—40 s, 72°—50 s)
*bla*_KPC_	KPC-F: ATGTCACTGTATCGCCGTCT KPC-R: TTTTCAGAGCCTTACTGCCC	883	30× (95°—30 s, 55°—40 s, 72°—50 s)
*bla*_VEB_	VEB-F:CGA CTT CCA TTT CCC GAT GC VEB-R: GGA CTC TGC AAC AAA TAC GC	643	30× (95°—30 s, 58.5°—40 s, 72°—50 s)
*bla*_GES_	GES-F: GTT TTG CAA TGT GCT CAA CG GES-R: TCG CAT AGC AAT AGG CGT AG	371	30× (95°—30 s, 56°—40 s, 72°—50 s)
*bla_PER_*	PER-F: AAT TTG GGC TTA GGG CAG AA PER-R: ATGAAT GTC ATT ATA AAA GC	925	30× (95°—30 s, 50°—40 s, 72°—50 s)
**Primers used for VM detection: *ompA; epsA; csuE; bap; bfmS***			
*ompA*	ompA-F: CGCTTCTGCTGGTGCTGAAT ompA-R: CGTGCAGTAGCGTTAGGGTA	531	30× (95°—30 s, 55°—40 s, 72°—50 s)
*epsA*	epsA-F: AGCAAGTGGTTATCCAATCG epsA-R: ACCAGACTCACCCATTACAT	451	
*bap*	bap-F: TACTTCCAATCCAATGCTAGGGAGGGTACCAATGCAG bap-R: TTATCCACTTCCAATGATCAGCAACCAAACCGCTAC	1225	35× (96°—60 s, 56.5°—60 s, 72°—60 s)
*csuE*	csuE-F: ATGCATGTTCTCTGGACTGATGTTGAC csuE-R: CGACTTGTACCGTGACCGTATCTTGATAAG	976	35× (96°—60 s, 57°—60 s, 72°—60 s)
*bfmS*	bfmS-F: TTGCTCGAACTTCCAATTTATTATAC bfmS-R: TTATGCAGGTGCTTTTTTATTGGTC	1368	35× (94°—60 s, 55°—60 s, 72°—45s)

**Table 2:** OXA-23- and -24-producing AB from Romanian hospitals and aquatic environments between 2019 and 2022.

Geographical Region	Isolation Sources (n)	Phenotype	β-Lactamases	VMs	Virulence Factors	Biofilm Production Capacity
S Romania (22)	IHI (3)	MDR (2)	OXA-23, OXA-24, OXA-51	*ompA*, *csuE*, *bfmS*, *bap*	lecithinase, amylase, caseinase	Moderate
					lecithinase, amylase, caseinase, lipase	Moderate
		Non-MDR (1)	OXA-24, OXA-51	*ompA*, *bfmS*	lecithinase, amylase, caseinase	Moderate
	SW (7)	MDR (3)	OXA-23, OXA-51	*ompA*, *csuE*, *bfmS*, *bap*	lecithinase, amylase, caseinase	Weak
					lecithinase, caseinase	Moderate
					lecithinase, amylase, caseinase, lipase	Weak
		MDR (2)	OXA-24, OXA-51, TEM	*ompA*, *csuE*, *bfmS*, *bap*	lecithinase, amylase, caseinase	Weak
						Moderate
		Non-MDR (2)	OXA-51	*ompA*, *bfmS*	lecithinase, caseinase	Strong
					lecithinase, amylase, caseinase, lipase	Moderate
	WW (15)	MDR (7)	OXA-23, OXA-51	*ompA*, *csuE*, *bfmS*, *bap*	lecithinase, amylase, caseinase	Weak
						Weak
						Weak
						Moderate
						Moderate
						Moderate
						Weak
		MDR (3)	OXA-24, OXA-51	*ompA*, *csuE*, *bfmS*	lecithinase, amylase, caseinase	Non-biofilm producer
					lecithinase, amylase, caseinase	Weak
					lecithinase, caseinase	Moderate
		MDR (3)	OXA-23, OXA-24, OXA-51	*ompA*, *csuE*	lecithinase, amylase, caseinase	Non-biofilm producer
					lecithinase, amylase, caseinase	Weak
					lecithinase, amylase	Weak
		MDR (2)	OXA-51		lecithinase, amylase, caseinase	Strong
					lecithinase, caseinase	Strong
N-E region (13)	SW (5)	MDR (3)	OXA-24, OXA-51, PER	*ompA*, *csuE*, *bfmS*, *bap*	lecithinase, amylase, caseinase	Weak
					lecithinase, amylase, caseinase	Weak
					lecithinase, amylase, caseinase, lipase	Moderate
		MDR (1)	OXA-24, OXA-51, TEM		lecithinase, amylase, caseinase, lipase	Weak
		Non-MDR (1)	OXA-51, TEM	*csuE*, *bfmS*	lecithinase, caseinase,	Moderate
	WW (8)	MDR (5)	OXA-23, OXA-51	*ompA*, *csuE*, *bfmS*	lecithinase, amylase, caseinase	Weak
					lecithinase, amylase, caseinase, lipase	Moderate
					lecithinase, amylase, caseinase, lipase	Moderate
					lecithinase, amylase, caseinase	Moderate
					lecithinase, amylase, caseinase	Moderate
		MDR (1)	OXA-24, OXA-51		lecithinase, caseinase, lipase	Moderate
		MDR (1)	OXA-24, OXA-51, TEM		lecithinase, amylase, caseinase	Moderate
		Non-MDR (1)	OXA-51	*ompA*, *bfmS*	lecithinase, amylase, caseinase, lipase	Strong
C-W Romania (9)	SW (4)	MDR (2)	OXA-23, OXA-51	*ompA*, *csuE*, *bfmS*, *bap*	lecithinase, amylase, caseinase, lipase	Weak
					lecithinase, amylase, caseinase	Non-biofilm producer
		Non-MDR (2)	OXA-51	*ompA*, *csuE*, *bfmS*	amylase, caseinase,	Non-biofilm producer
					lecithinase, amylase, caseinase	Strong
	WW (5)	MDR (1)	OXA-24, OXA-51	*ompA*, *csuE*, *bfmS*	lecithinase, caseinase, lipase	Non-biofilm producer
		MDR (2)	OXA-23, OXA-51	*ompA*, *csuE*, *bfmS*	lecithinase, caseinase, lipase	Non-biofilm producer
					lecithinase, amylase, lipase	Moderate
		Non-MDR (2)	OXA-51	*csuE*, *bfmS*	lecithinase, amylase, caseinase, lipase	Moderate
					lecithinase, caseinase, lipase	Weak

n—number of isolates.

**Table 3:** The influence of Ag NPs on the secretion of hydrolytic enzymes associated with virulence and pathogenicity.

			Caseinase Activity (%)			Lipase Activity (%)		
Region	Isolation Source	Strain	Control	MIC/2	*p*-Value	Control	MIC/2	*p*-Value
N-E region	WW	4	100 ± 16.6	127.77 ± 9.6	0.5785	-	-	-
		18	100 ± 14.2	109.52 ± 29.7	>0.9999	-	-	-
		19	100 ± 8.6	95 ± 31.2	>0.9999	100 ± 21.65	100 ± 21.65	>0.9999
		29	100 ± 16.3	82.14 ± 16.3	0.9975	100 ± 34.64	80 ± 34.64	>0.9999
		30	100 ± 11.1	40.74 ± 6.4	<0.0001	100 ± 43.3	0 ± 0	0.0069
		31	100 ± 18.3	100 ± 6.9	>0.9999	100 ± 24.74	57.14 ± 24.74	0.9254
		33	100 ± 6.92	40 ± 6.9	<0.0001	-	-	-
	SW	32	100 ± 11.1	100 ± 11.1	>0.9999	-	-	-
		1	100 ± 10.18	100 ± 20.37	>0.9999	100 ± 21.65	75 ± 37.5	>0.9999
		2	100 ± 16.6	111.11 ± 9.62	>0.9999	-	-	-
		3	100 ± 14.2	104.76 ± 8.24	>0.9999	-	-	-
		35	100 ± 7.35	104.34 ± 0	>0.9999	-	-	-
		44	100 ± 20	0 ± 0	<0.0001	100 ± 0	0 ± 0	0.0069
S region	SW	5	100 ± 10.1	105.88 ± 17.6	>0.9999	-	-	-
		7	100 ± 27.1	73.91 ± 7.53	0.7113	100 ± 0	88,88 ± 38.4	>0.9999
		12	100 ± 12.5	95.83 ± 7.53	>0.9999	-	-	-
		13	100 ± 24.7	104.76 ± 8.24	>0.9999	-	-	-
		20	100 ± 10.8	112.5 ± 18.75	>0.9999	100 ± 13.8	96 ± 12	>0.9999
		21	100 ± 6.6	96.15 ± 6.66	>0.9999	-	-	-
	WW	22	100 ± 7.5	91.30 ± 13.3	>0.9999	-	-	-
		34	100 ± 5.2	140 ± 20	0.039	-	-	-
		36	100 ± 14.2	90.47 ± 16.49	>0.9999	-	-	-
		37	100 + 8.6	100 ± 8.66	>0.9999	-	-	-
		14	100 ± 7.5	95.65 ± 7.53	>0.9999	-	-	-
		39	100 ± 0	116.66 ± 16.6	0.9993	-	-	-
		40	100 ± 7.8	95.45 ± 7.53	>0.9999	-	-	-
		41	100 ± 28.6	118.75 ± 10.8	0.994	100 ± 24.74	85.71 ± 0	>0.9999
		42	100 ± 10.8	118.75 ± 10.8	0.994	-	-	-
		43	100 ± 6.6	92.30 ± 0	>0.9999	-	-	-
		6	100 ± 12.5	91.66 ± 26.0	>0.9999	-	-	-
		8	100 ± 0	91.66 ± 7.21	>0.9999	-	-	-
	IHI	9	100 ± 19.92	95.65 ± 7.53	>0.9999	100 ± 34.6	180 ± 0	0.0739
		11	100 ± 7.53	95.65 ± 7.53	>0.9999	-	-	-
		38	100 ± 12.5	91.66 ± 7.21	>0.9999	-	-	-
C-W region	WW	16	100 ± 7.87	109.09 ± 1.75	>0.9999	100 ± 34.6	60 ± 0	0.9608
		17	100 ± 7.53	86.95 ± 7.53	>0.9999	100 ± 34.6	60 ± 0	0.9608
		28	100 ± 15.06	100 ± 7.53	>0.9999	100 ± 15.74	109.09 ± 1.74	>0.9999
		25	-	-		100 ± 0	66.66 ± 14.43	0.995
	SW	23	100 ± 12.5	87.5 ± 12.5	>0.9999	-	-	-
		24	100 ± 7.53	82.60 ± 7.53	0.9985	-	-	-
		26	100 ± 18.23	131.57 ± 9.11	0.3033	100 ± 17.32	60 ± 30	0.9608
		15	100 ± 6.66	84.61 ± 17.62	0.9999	-	-	-

“-” refers to non-secreting strains (Dunn–Šidák method).

**Table 4:** The percentage of NO consumed from the total identified in the control samples.

Sample	The Percentage of NO Consumed (%)
30	12.93 ± 1.46
12	9.23 ± 3.92
11	23.20 ± 5.67
31	50.99 ± 2.68
6	28.58 ± 0.44
43	44.75 ± 3.87
32	20.48 ± 6.22
5	0.44 ± 9.51
33	14.08 ± 2.08
44	11.85 ± 1.71

## Data Availability

Samples of the compounds, AB isolates and data sets regarding the results are available from the authors.

## Supplementary Materials

The following supporting information can be downloaded at https://www.mdpi.com/article/10.3390/microorganisms11102439/s1. Figure S1: Disk diffusion screening assays of the antimicrobial activity of the AgNPs (1-AgNPc; 2- AgNPt; 3-AgNPsol; 4-AuNP; 5-CuNP; 6-ZnONP) against A. baumannii strains isolated in 2019 (a); 2020 (b); 2021 (c) and from WW and SW samples.; Figure S2: Electrophoresis gel for blaOXA-51 (353 bp) gene detection; Figure S3: Electrophoresis gel for blaOXA-23 (501 bp) gene detection; Figure S4: Electrophoresis gel for blaOXA-23 (501 bp) and blaOXA-24 (242 bp) genes detection; Figure S5: Electrophoresis gel for blaPER (925 bp) gene detection; Figure S6: Electrophoresis gel for blaTEM (1080 bp) gene detection; Figure S7: Electrophoresis gel for ompA (531 bp) gene detection; Figure S8: Electrophoresis gel for ompA (531 bp) gene detection; Figure S9: Electrophoresis gel for bfmS (1368 bp) gene detection; Figure 10: Electrophoresis gel for csuE (976 bp) gene detection; Figure S11: Electrophoresis gel for csuE (976 bp) gene detection; Figure S12: Disk diffusion screening assays of the synergic antimicrobial activity of the Ag NPsol and antibiotics (IMP, SAM, MEM, CAZ, ATM, FEP, CN, DOR, CIP and AK) against A. baumannii strains isolated in 2020 from WW samples (N-E Romania); Table S1: A. baumannii strains investigated in the study; Table S2: Summary of the interaction of the AgNP and the various antibiotics.
