# Supplementary material for: Phenotypic and Genotypic Characterization of Recently Isolated Multidrug-Resistant Acinetobacter baumannii Clinical and Aquatic Strains and Demonstration of Silver Nanoparticle Potency
Source: Microorganisms. 2023 Sep 28;11(10):2439. doi: 10.3390/microorganisms11102439 (PMC10609299; doi:10.3390/microorganisms11102439)
Supplement: Supplementary file 1 [file microorganisms-11-02439-s001.zip › microorganisms-2609891-supplementary.pdf]

microorganisms

MDPI

Supplementary Material

Phenotypic and genotypic characterization of recently isolated multidrug resistant *Acinetobacter baumannii* clinical and aquatic strains and demonstration of silver nanoparticles potency

Irina Gheorghe-Barbu †[1,2], Viorica Maria Corbu*[1,2], Corneliu Ovidiu Vrancianu †[1,2,3], Ioana Cristina Marinas †[1], Marcela Popa[1,2] Andreea Ștefania Dumbravă[1,2], Mihai Niță-Lazar[4] Ionut Pecete[5], Andrei Alexandru Muntean[6,7], Mircea Ioan Popa[6,7], Liliana Marinescu[8], Denisa Ficai[8], Anton Ficai[8] and Ilda Czobor Barbu[1,2]

S1. Supplementary Figures and Tables

S1.1. Supplementary Figures

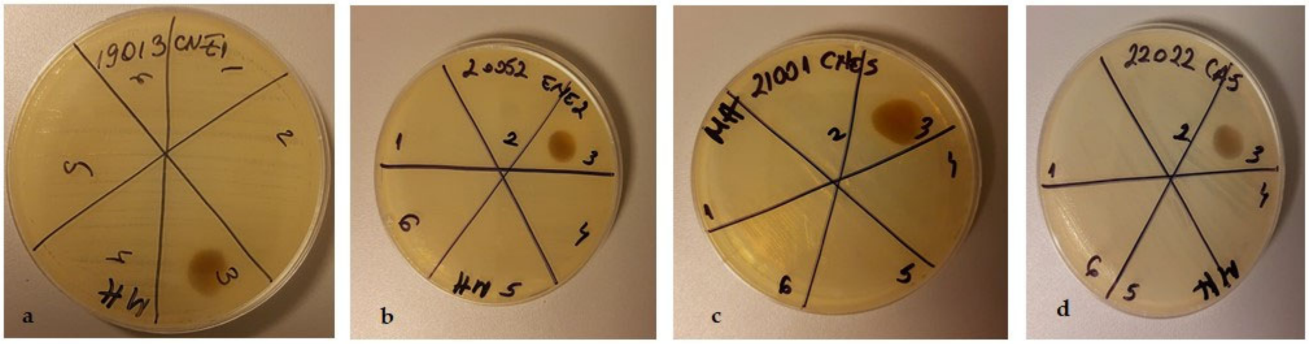

Supplementary Figure S1. Disk diffusion screening assays of the antimicrobial activity of the AgNPs ((1-AgNPc; 2-AgNPt; 3-AgNPsol; 4-AuNP; 5-CuNP; 6-ZnONP) against *A. baumannii* strains isolated in 2019 (a); 2020 (b); 2021 (c) and from WW and SW samples.

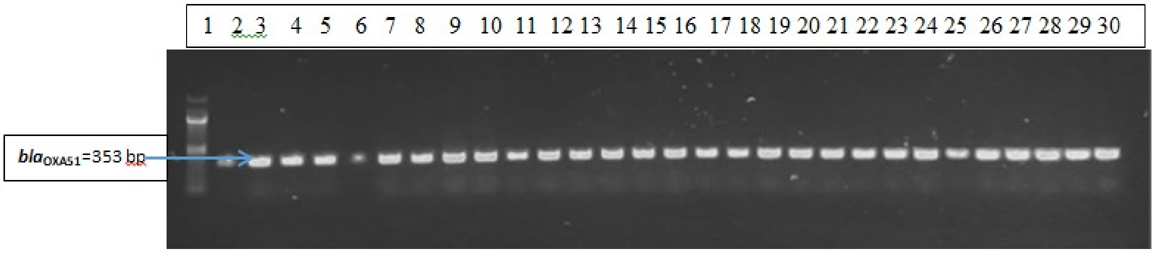

Supplementary Figure S2. Electrophoresis gel for bla_{OXA-51} (353 bp) gene detection: all *A. baumannii* strains were positive. Lines: **1**- Molecular Size Marker (ThermoScientific, 1500 bp); **2-** strain 19012 ENE2; **3**- 19042 CNE3; **4**-111; **5**-21018 CNE6; **6-** 21010 ONE3; **7**-22014CA2; **8**-19015 CNE2; **9**-19013 ONE1; **10-** 19014 CNE2; **11**- 20031 ONE5; **12**-22019CNE5; **13**-22018 CA6; **14**-20030 ONE3; **15**-21045 CNE6; **16**-21049 ENE3; **17**-21012 CNE5; **18**-22012 CA5; **19**-20070 ENE4; **20-**19059 ENE1; **21**-19060 ENE2; **22**- 20070 ENE4; **23**-Ab62 VL; **24**-21002 CNE4; **25**-21001 CNE5; **26**-22007 CA4; **27**-19022 CNE4; **28**-22025 CA5; **29**-Ab82 VL; 30- *A. baumannii* positive control for bla_{OXA-51} gene.

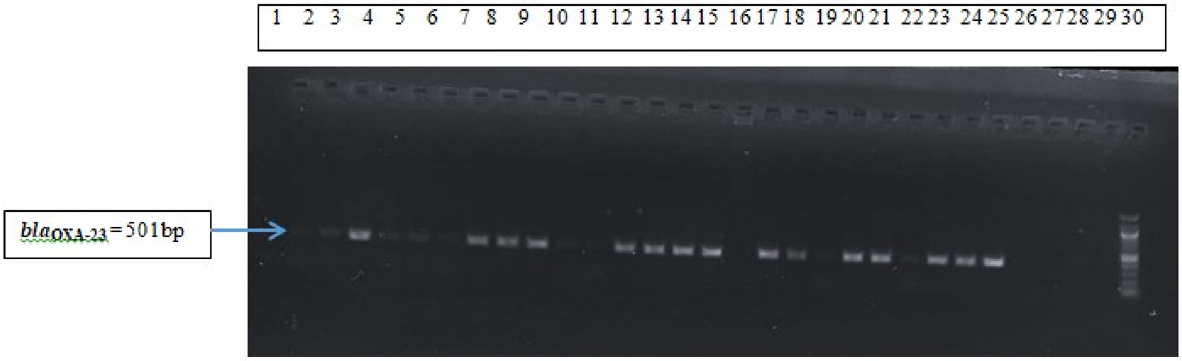

Supplementary Figure S3. Electrophoresis gel for *bla*$_{OXA-23}$ (501 bp) gene detection: 20 *A. baumannii* strains were positive. Lines: **2-** strain 19012 ENE2; **3-** 111; **4**-21010 ONE3; **5**- 19015 CNE2; **6**-19013 ONE1; **7**-19014 CNE2; **8-**20031 ONE5; **9**-21045 CNE6; **10**-21012 CNE5; **11**-19045 ENE5; **12**-21002 CNE4; **13**-21001 CNE5; **14-**20056 CNE2; **15**-20113 ENE3; **16-**22018 CA6; **17**-19003 CNE1; **18**-21036 CNE4; **19**-22004 CA3; **20**-22003 ENE2; **21**-22014 CA2; **22**-22025 CA5; **23**-21018 CNE6; **24**-20070 ENE4; **25**- 19094 CNE1; **26**-22004 CA3; **27**-21030 ENE4; **28**-22006 CA3; **29**-19051 ENE2; **30** –negative control; Molecular Size Marker (ThermoScientific, 1500 bp).

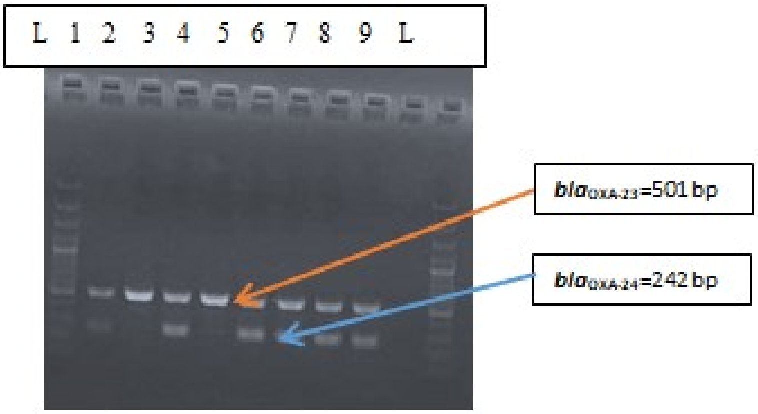

Supplementary Figure S4. Electrophoresis gel for *bla*$_{OXA-23}$ (501 bp) and *bla*$_{OXA-24}$ (242 bp) genes detection: 5 strain of *A. baumannii* encoded BL3Aba11; 30 Trg; 111; 21018 CNE6; 22014 CA2 were positives. Lines: **1**- Molecular Size Marker (ThermoScientific, 1500 bp); **2-** strain BL3Aba11; **3-** strain 22018 CA6; **4-** strain 30 Trg; ; **5-** 19060 ENE2; **6-** 111; **7-**21018 CNE6; **8**- 22014 CA2; 9- *A. baumannii* positive control for *bla*$_{OXA-23}$ and *bla*$_{OXA-24}$ genes.

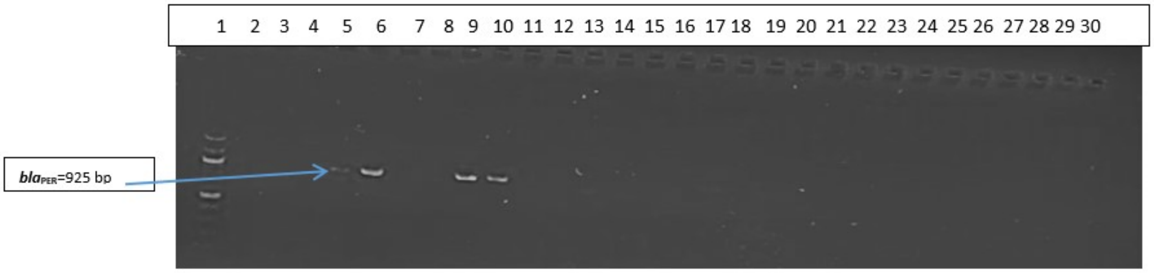

Supplementary Figure S**5**. Electrophoresis gel for *bla*$_{PER}$ (925 bp) gene detection: 3 strain of *A. baumannii* encoded 21039 CNE5; 21030 ENE4; 21030 CNE3 were positive. Lines: **1**- Molecular Size Marker (ThermoScientific, 1500 bp); **2-** strain

22025 CA5; **3-** strain 22028 CNE2; **4-** strain 19001 CNE1; ; **5-** 21039 CNE5; **6**-21030ENE4; **7-** 19094 CNE6; **8**-19094 CNE5; **9**-21030 CNE3; **10**- *A. baumannii* positive control for bla_{PER} gene; **11**- 19003 CNE1; **12**- 21036 CNE4; **13**- 22028 CNE6; **14**-19091 ENE4; **15**- 19094 CNE1; **16**- 19094 ENE5; **17**- 22003 ENE2; **18**- 22002 COLN3; **19**- 19005 CNE2; **20**- 19001 CNE1; **21**-20018 ENE4; **22**- DF 0965;**23**- DF 0462; **24**- 19052 ENE2; **25**- 21002 CNE2; **26**- 20029 ONE2; **27**- 22019 CNE4; **28**- 19018 CNE2; **29**- 20029 ONE2; 30-negative control.

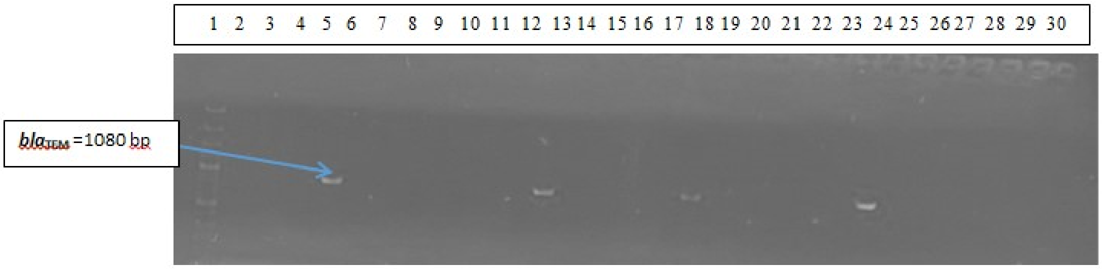

Supplementary Figure S**6.** Electrophoresis gel for bla_{TEM} (1080 bp) gene detection: 3 strains of *A. baumannii* encoded 22025 CA5; 22026 CNE1; 19016 ONE4 were positive. Lines: **1**- Molecular Size Marker (ThermoScientific, 1500 bp); **2-** strain 20018 CNE3; **3**-20015 CNE3; **4**-20085 CNE5; **5**- 22025 CA5; **6**-20084 CNE2; **7**-20018 ENE4; **8**-21035 CNE6;**9**-21036 ENE2; **10**-21036 ONE3;**11**-21036 CNE4; **12**-22026 CNE1; **13**-21039 CNE5;**14**-22028 CNE2;**15**-22028 ENE5; **16-**19016 ONE3; **17**-20034 CNE4; **18-**19016 ONE4; **19**-21020 CNE1; **20**-22019 CNE5; **21**-BL3Aba 5; **22**-19044 CNE6; **23**-21012 CNE3; **24**- *A. baumannii* positive control for bla_{TEM} gene; **25**- 21017 CNE3; **26**- 22015 CA3; 20070 ENE4; **27**- 19022 CNE4; **28-** 22022 CA5; **29-**20056 CNE2; 30- negative control.

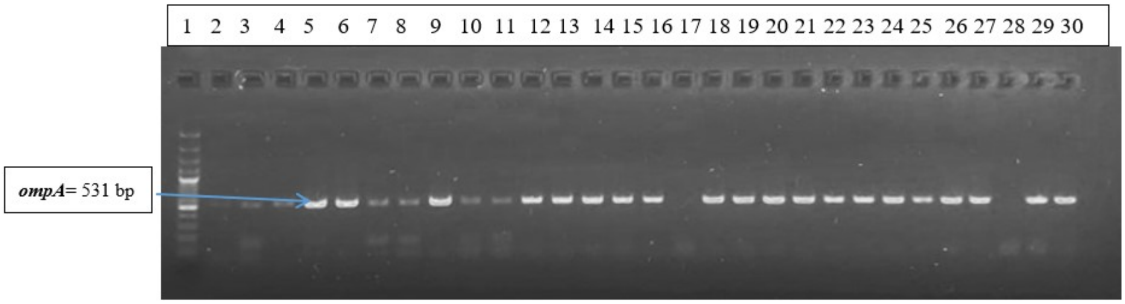

Supplementary Figure S7. Electrophoresis gel for *ompA* (531 bp) gene detection: 26 *A. baumannii* strains were positive.Lines: **1**- Molecular Size Marker (ThermoScientific, 1500 bp); **2-** strain BL3Aba5; **3**-30 Trg; **4**-Ab 62 VL; **5**-21012 CNE5; **6**-21012 CA5; **7**-20070 ENE4; **8**-20069 ENE1; **9**-19059 ENE1; **10**-19060 ENE2; **11**-19045 ENE5; **12**-19012 ENE2; **13**—19042 CNE3; **14**-111; **15**-21018 CNE6; **16**-22026 CNE1; **17** – 21049 ENE3; **18**- 21010 ONE3; **19**-22014 CA2; **20**-19015 CNE2; **21**-19013 ONE1; **22**-19014 CNE2; **23**-20031 ONE5; **24**-22019 CNE5; **25**-22018 CA6; **26**-20030 ONE3; **27**- 21045 CNE6; **28**-22025 CA5; **29**-21039 CNE5; 30- *A. baumannii* positive control for *ompA* gene.

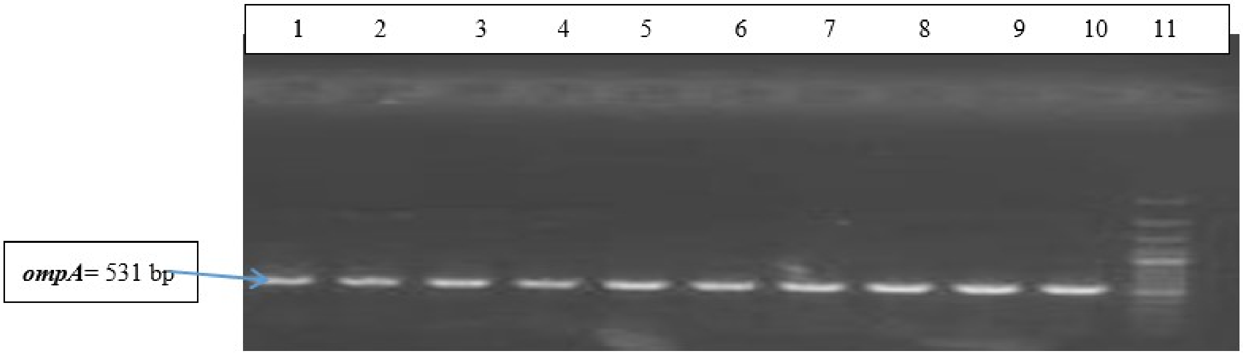

Supplementary Figure S8. Electrophoresis gel for *ompA* (531 bp) gene detection: 9 *A. baumannii* strains were positive. Lines: **1**-strain 19003 CNE1; **2**-20085 CNE3; **3**-21036 CNE4; **4**-22028 CNE6; **5**-19094 CNE6; **6**- 22003 ENE2; **7**- 19051 ENE2; **8**-22006 CA3; **9**- 21001 CNE5; 10-*A. baumannii* positive control for *ompA* gene; 11- Molecular Size Marker (ThermoScientific, 1500 bp).

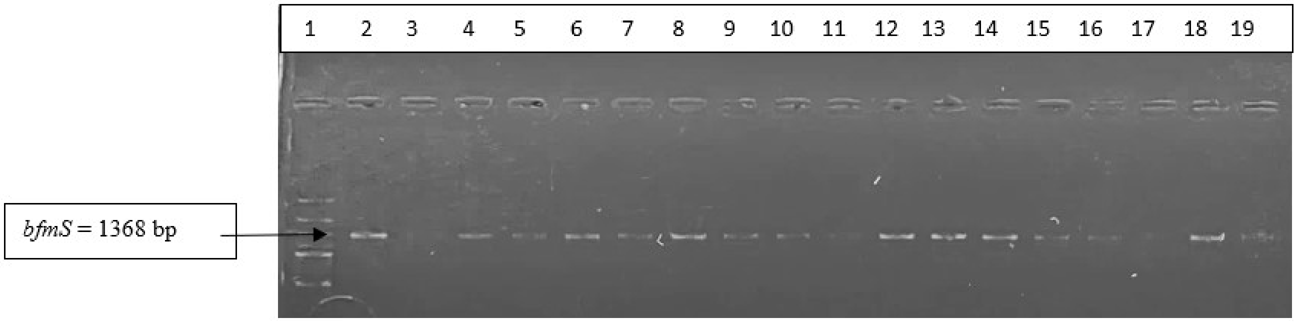

Supplementary Figure S9. Electrophoresis gel for *bfmS* (1368 bp) gene detection: 15 *A. baumannii* strains were positive. Lines: **1**- Molecular Size Marker (ThermoScientific, 1500 bp); **2-** strain BL3Aba5; **3**-30 Trg; **4**-Ab 62 VL; **5**-21012 CNE5; **6**-22012 CA5; **7**-20070 ENE4; **8**-20069 ENE1; **9**-19059 ENE1; **10**-19060 ENE2; **11**-19045 ENE5; **12**-19012 ENE2; **13**—19042 CNE3; **14**-111; **15**-21018 CNE6; 16-21010 ONE3; 17- 22014 CA2; 18- *A. baumannii* positive control for *bfmS* gene; 19-19015 CNE2.

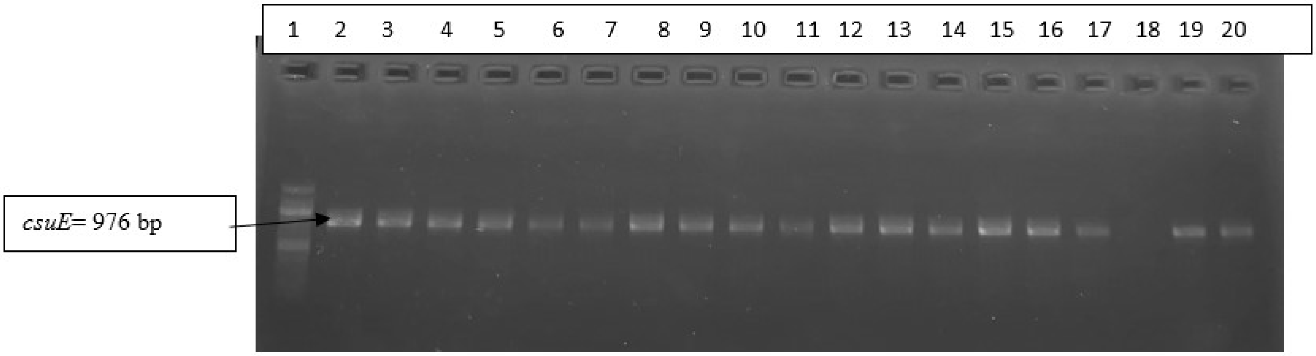

Supplementary Figure S10. Electrophoresis gel for *csuE* (976 bp) gene detection: 18 *A. baumannii* strains were positive. Lines: 1- Molecular Size Marker (ThermoScientific, 1500 bp); **2-** strain BL3Aba5; **3**-21012 CNE5; **4**-22012 CA5; **5**-20070 ENE4; **6**-20069 ENE1; **7**- 19059 ENE1; **8**-19060 ENE2; **9**-19045 ENE5; **10**-19012 ENE2; **11**-19042 CNE3; **12**-21018 CNE6; **13**-21010 ONE3; **14**-21010 ONE3;**15**-22014 CA2; **16**-19015 CNE2; **17**-19013 ONE1; **18**-111; **19**-19014 CNE2; 20- *A. baumannii* positive control for *csuE* gene.

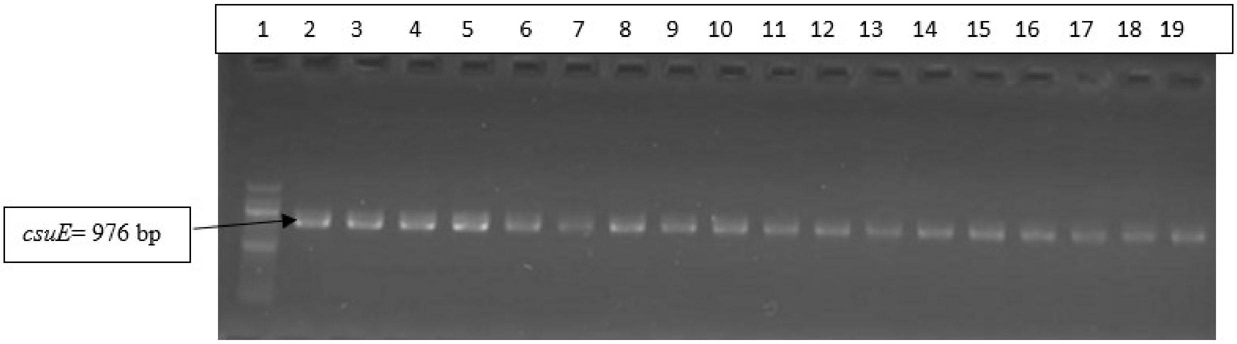

Supplementary Figure S11. Electrophoresis gel for *csuE* (976 bp) gene detection: 17 *A. baumannii* strains were positive. Lines: 1- Molecular Size Marker (ThermoScientific, 1500 bp); **2-**20031 ONE5; **3**-22019 CNE5; **4-**22018 CA6; **5**-22025 CA5; **6**-21039 CNE5; **7**-22026 CNE1; **8**-21030 ENE4; **9**- 19003 CNE1; **10**-21036 CNE4; **11**-22028 CNE6; **12**-19091 ENE4; **13**-22003 ENE2; **14**-19051 ENE2; **15**-20056 CNE2; **16**-22006 CA3; **17**-20113 ENE3; **18**-22022 CA5; 19- *A. baumannii* positive control for *csuE* gene.

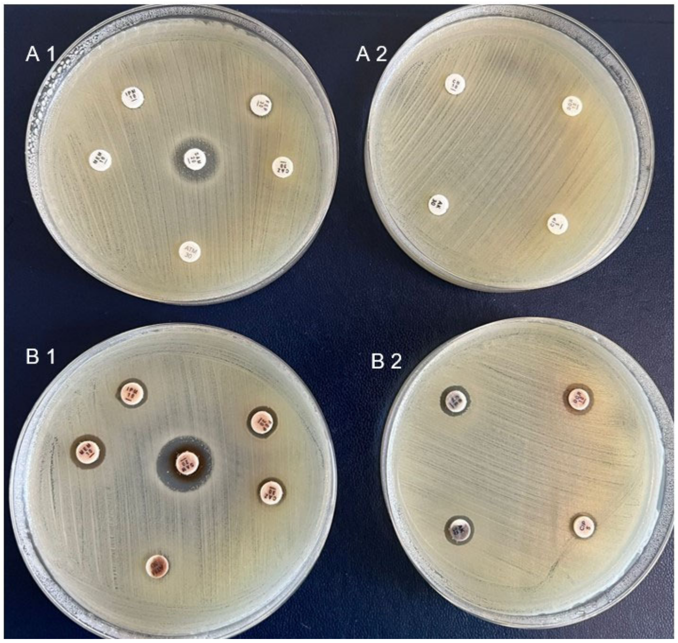

Supplementary Figure S12. Disk diffusion screening assays of the synergic antimicrobial activity of the Ag NPsol and antibiotics (IMP, SAM, MEM, CAZ, ATM, FEP, CN, DOR, CIP and AK) against *A. baumannii* strains isolated in 2020 from WW samples (N-E Romania): A1 and A2 correspond to 20085 CNE5 strain disc diffusion assay and respectively B1 and B2 to synergistic activity between antibiotics and Ag NPsol (5 µL).

S1.2. Supplementary Tables

Supplementary Table S1. ***A. baumannii*** **strains investigated in the study.**

Strain code	Isolation source	Year of isolation	Geographical region, location	Strain code	Isolation source	Year of isolation	Geographical region, location
30 TRG	IHI	2019	Southern, Targoviste	19094 ENE5	WWs	2019	North-Eastern, Iasi
80 TRG				19091 ENE4			
19016 ONE4 (T19)	SWs			19094 CNE1			
19016 ONE5 (T20)				19094 CNE6			
19016 ONE3				19094 CNE5			
19016 CNE2				88 Ab Iasi (Ab 26)	IHI	2020	
19015 CNE2	WWs			20006 CNE6 (Ab13)	WWs		
19013 ONE1				20096 CNE2 (Ab28)			
19015 CNE6				20096 ONE4 (Ab35)			
19013 CNE1				21030 CNE3 (Ab14)	SWs	2021	
19014 CNE1				21030 ENE4 (Ab 13)			
19014 CNE4				22003 ENE2	WWs	2022	
19018 CNE2				22002 CA5	SWs		
19018 CNE4				22002 COLN3			
19014 ENE4				19005 CNE2	SWs	2019	North-Eastern, Galati
20034 CNE4 (T72)	WWs	2020		19003 CNE6	WWs		
20029 ENE1 (T38)				19003 CNE1			
20029 CNE2 (T45)				19006 CNE5			
20029 ONE2 (T42)				19001 CNE1			
21019 ONE2 (T26)	WWs	2021		19006 ONE1			
21020 CNE1 (T23)				19001 ONE1			
21020 ONE3 (T22)				20018 CNE3 (Ab95)	WWs	2020	
21020 CNE5 (T21)				20015 CNE3 (Ab91)			
22019 CNE5	WWs	2022		20085 CNE5 (Ab113)			
22018 CA5				20084 CNE2 (Ab109)			
22018 CA6				20018 ENE4 (Ab93)			
22019 CNE4				21035 CNE6	WWs	2021	
BL3Aba 5	IHI	2019	Southern, Bucharest	21036 ENE2			
BL3Aba 11				21036 ONE3			
19045 ENE5	SWs			21036 CNE4			
19045 ENE2				21039 CNE5	SWs		
19044 CNE6				22025 CA5	SWs	2022	
19044 CNE5				22028 CNE2	WWs		
19042 CNE6	WWs			22028 ENE5			
19011 CNE6				22028 CNE6			
19012 ENE2	WWs			DF 0965	IHI		Central-Western, Cluj
19042 CNE3				DF 0462			

111				19051 ENE2	SWs	2019	
108	IHI	2020		19049 ENE4			
110				19049 ENE1	WWs		
109				19052 ENE2			
21012 CNE3 (Ab50)				20056 CNE2	SWs		
21012 CNE5 (Ab 51)	SWs			20056 ENE4			
21012 ONE2 (Ab53)				20054 CNE4			
21010 ONE1				20052 ENE2			
21018 CNE6		2021		20103 ENE4		2020	
21017 CNE3				20039 ONE1	WWs		
21010 CNE3	WWs			20039 CNE5			
21018 CNE1				20104 CNE5			
21009 CNE1				21002 CNE4			
22012 ENE6	SWs			21001 CNE1	WWs	2021	
22012 CA5				21002 CNE2			
22013 ENE4				21001 CNE5			
22014 ENE5				22005 ENE1			
22014 CA2		2022		22005 ENE4	SWs		
22015 CA4	WWs			22005 CA3			
22015 CA3				22006 CA2			
22013 CA5				22007 CA3		2022	
Ab62 VL	IHI			22007 CA2	WWs		
Ab82 VL				22007 CA4			
19059 ENE1	SWs			19022 CNE6	WWs		
19060 ENE2		2019		19022 CNE4		2019	Central-Western, Timisoara
19058 ENE1	WWs		Southern, Valcea	20113 ENE3	SWs	2020	
19062 ENE1				22022 CA5	WWs	2022	
20070 ENE4 (Ab 60)	SWs						
20069 ENE1 (Ab 59)		2020					
20073 ENE3 (Ab 68)	WWs						
21049 ENE3	WWs	2021					

Supplementary Table S2. Summary of the interaction of the Ag NPsol and the various antibiotics.

Laboratory code	Isolation code	Geographical region	Antibiotics	Antibiotic alone (mm)	Antibiotic with Ag NP (mm)	% change in IZD	Interpretation
18	21036CNE4	WWs N-E	IMP	6	9	50	Synergism
			SAM	6	12	100	Synergism
			MEM	6	11	83.33	Synergism
			CAZ	6	8	33.33	Synergism
			ATM	15	15	0	Indifference
			FEP	10	10	0	Indifference
			CN	6	6	0	Indifference

			DOR	6	6	0	Indifference
			CIP	6	11	83.33	Synergism
			AK	6	7	16.67	Additive
19	20085 CNE3	WWs N-E	IMP	6	8	33.33	Synergism
			SAM	6	11	83.33	Synergism
			MEM	6	10	66.67	Synergism
			CAZ	6	8	33.33	Synergism
			ATM	6	6	0	Indifference
			FEP	6	9	50	Synergism
			CN	6	9	50	Synergism
			DOR	6	9	50	Synergism
			CIP	6	7	16.67	Additive
			AK	6	8	33.33	Synergism
4	22028CNE4	WWs N-E	IMP	6	9	50	Synergism
			SAM	14	14	0	Indifference
			MEM	6	9	50	Synergism
			CAZ	12	14	16.67	Additive
			ATM	15	16	6.67	Additive
			FEP	15	15	0	Indifference
			CN	6	8	33.33	Synergism
			DOR	6	6	0	Indifference
			CIP	6	6	0	Indifference
			AK	9	11	22.22	Synergism
27	19003CNE1	WWs N-E	IMP	6	8	33.33	Synergism
			SAM	6	14	133.33	Synergism
			MEM	6	8	33.33	Synergism
			CAZ	6	8	33.33	Synergism
			ATM	12	13	8.33	Additive
			FEP	10	11	10	Additive
			CN	6	7	16.67	Additive
			DOR	6	8	33.33	Synergism
			CIP	6	6	0	Indifference
			AK	6	6	0	Indifference
33	22003ENE2	WWs N-E	IMP	6	8	33.33	Synergism
			SAM	6	13	116.67	Synergism
			MEM	6	8	33.33	Synergism
			CAZ	14	10	-28.57	Antagonism
			ATM	14	13	-7.14	Antagonism
			FEP	11	12	9.09	Additive
			CN	6	7	16.67	Additive
			DOR	6	8	33.33	Synergism
			CIP	6	7	16.67	Additive
			AK	6	7	16.67	Additive
29	19094CNE6	WWs N-E	IMP	6	7	16.67	Additive
			SAM	11	13	18.18	Additive
			MEM	6	7	16.67	Additive
			CAZ	6	11	83.33	Synergism
			ATM	13	16	23.08	Synergism
			FEP	15	14	-6.67	Antagonism
			CN	6	7	16.67	Additive
			DOR	6	7	16.67	Additive
			CIP	6	7	16.67	Additive
			AK	7	10	42.86	Synergism
30	22004CA3	WWs N-E	IMP	32	34	6.25	Additive
			SAM	30	40	33.33	Synergism

			MEM	30	32	6.67	Additive
			CAZ	24	24	0	Indifference
			ATM	19	24	26.32	Synergism
			FEP	24	30	25	Synergism
			CN	20	21	5	Additive
			DOR	30	31	3.33	Additive
			CIP	28	31	10.71	Additive
			AK	20	20	0	Indifference
31	19091ENE4	WWs N-E	IMP	32	34	6.25	Additive
			SAM	30	30	0	Indifference
			MEM	30	30	0	Indifference
			CAZ	24	24	0	Indifference
			ATM	19	18	-5.26	Antagonism
			FEP	28	26	-7.14	Antagonism
			CN	19	21	10.53	Additive
			DOR	28	28	0	Indifference
			CIP	28	29	3.57	Additive
			AK	19	20	5.26	Additive
1	21039CNE 5	SWs N-E	IMP	6	8	33.33	Synergism
			SAM	6	6	0	Indifference
			MEM	6	10	66.67	Synergism
			CAZ	6	10	66.67	Synergism
			ATM	12	11	-8.33	Antagonism
			FEP	6	9	50	Synergism
			CN	6	8	33.33	Synergism
			DOR	6	9	50	Synergism
			CIP	6	9	50	Synergism
			AK	9	10	11.11	Additive
2	22026CNE1	SWs N-E	IMP	6	9	50	Synergism
			SAM	14	13	-7.14	Antagonism
			MEM	6	10	66.67	Synergism
			CAZ	6	8	33.33	Synergism
			ATM	14	13	-7.14	Antagonism
			FEP	15	8	-46.67	Antagonism
			CN	6	9	50	Synergism
			DOR	6	7	16.67	Additive
			CIP	6	8	33.33	Synergism
			AK	8	11	37.5	Synergism
3	22025CA5	SWs N-E	IMP	34	32	-5.88	Antagonism
			SAM	24	30	25	Synergism
			MEM	28	30	7.14	Additive
			CAZ	24	25	4.17	Additive
			ATM	20	20	0	Indifference
			FEP	26	26	0	Indifference
			CN	21	21	0	Indifference
			DOR	26	26	0	Indifference
			CIP	26	29	11.54	Additive
			AK	20	21	5	Additive
32	21030CNE3	SWs N-E	IMP	6	7	16.67	Additive
			SAM	19	20	5.26	Additive
			MEM	6	7	16.67	Additive
			CAZ	6	7	16.67	Additive
			ATM	15	15	0	Indifference
			FEP	16	15	-6.25	Antagonism
			CN	8	9	12.5	Additive
			DOR	6	8	33.33	Synergism

			CIP	6	7	16.67	Additive
			AK	6	7	16.67	Additive
44	21030ENE4	SWs N-E	IMP	11	11	0	Indifference
			SAM	26	29	11.54	Additive
			MEM	9	10	11.11	Additive
			CAZ	20	21	5	Additive
			ATM	17	19	11.77	Additive
			FEP	22	22	0	Indifference
			CN	17	21	23.53	Synergism
			DOR	8	10	25	Synergism
			CIP	27	27	0	Indifference
			AK	14	19	35.71	Synergism
9	BL3ABA5	IHI S	IMP	6	10	66.67	Synergism
			SAM	6	8	33.33	Synergism
			MEM	6	9	50	Synergism
			CAZ	6	8	33.33	Synergism
			ATM	12	8	-33.33	Antagonism
			FEP	6	8	33.33	Synergism
			CN	6	9	50	Synergism
			DOR	6	8	33.33	Synergism
			CIP	6	7	16.67	Additive
			AK	6	7	16.67	Additive
11	62Valcea spital	IHI S	IMP	6	9	50	Synergism
			SAM	13	15	15.39	Additive
			MEM	6	8	33.33	Synergism
			CAZ	6	8	33.33	Synergism
			ATM	10	8	-20	Antagonism
			FEP	6	8	33.33	Synergism
			CN	6	7	16.67	Additive
			DOR	6	7	16.67	Additive
			CIP	6	7	16.67	Additive
			AK	6	7	16.67	Additive
38	30SPIT TRG	IHI S	IMP	6	8	33.33	Synergism
			SAM	16	16	0	Indifference
			MEM	6	7	16.67	Additive
			CAZ	6	6	0	Indifference
			ATM	14	16	14.29	Additive
			FEP	12	14	16.67	Additive
			CN	6	8	33.33	Synergism
			DOR	6	8	33.33	Synergism
			CIP	6	7	16.67	Additive
			AK	8	9	12.5	Additive
10	21018CNE6	WWs S	IMP	6	9	50	Synergism
			SAM	6	9	50	Synergism
			MEM	6	10	66.67	Synergism
			CAZ	6	9	50	Synergism
			ATM	10	8	-20	Antagonism
			FEP	6	9	50	Synergism
			CN	6	8	33.33	Synergism
			DOR	6	7	16.67	Additive
			CIP	6	7	16.67	Additive
			AK	6	6	0	Indifference
14	21045CNE6	WWs S	IMP	6	9	50	Synergism
			SAM	6	10	66.67	Synergism
			MEM	6	8	33.33	Synergism

			CAZ	6	7	16.67	Additive
			ATM	8	8	0	Indifference
			FEP	6	7	16.67	Additive
			CN	6	6	0	Indifference
			DOR	6	7	16.67	Additive
			CIP	6	6	0	Indifference
			AK	6	6	0	Indifference
22	21049ENE3	WWs S	IMP	6	9	50	Synergism
			SAM	13	13	0	Indifference
			MEM	6	9	50	Synergism
			CAZ	6	8	33.33	Synergism
			ATM	15	7	-53.33	Antagonism
			FEP	6	9	50	Synergism
			CN	6	8	33.33	Synergism
			DOR	6	9	50	Synergism
			CIP	6	8	33.33	Synergism
			AK	6	9	50	Synergism
34	19015CNE2	WWs S	IMP	6	8	33.33	Synergism
			SAM	6	9	50	Synergism
			MEM	6	8	33.33	Synergism
			CAZ	12	10	-16.67	Antagonism
			ATM	16	15	-6.25	Antagonism
			FEP	12	12	0	Indifference
			CN	6	6	0	Indifference
			DOR	6	8	33.33	Synergism
			CIP	6	6	0	Indifference
			AK	6	6	0	Indifference
35	20030ONE1	WWs S	IMP	6	9	50	Synergism
			SAM	6	9	50	Synergism
			MEM	6	8	33.33	Synergism
			CAZ	6	7	16.67	Additive
			ATM	9	9	0	Indifference
			FEP	8	7	-12.5	Antagonism
			CN	6	7	16.67	Additive
			DOR	6	7	16.67	Additive
			CIP	6	7	16.67	Additive
			AK	6	7	16.67	Additive
36	22018CA6	WWs S	IMP	30	30	0	Indifference
			SAM	30	32	6.67	Additive
			MEM	26	32	23.08	Synergism
			CAZ	22	24	9.09	Additive
			ATM	22	24	9.09	Additive
			FEP	24	30	25	Synergism
			CN	18	20	11.11	Additive
			DOR	28	30	7.14	Additive
			CIP	30	30	0	Indifference
			AK	16	16	0	Indifference
37	22014CA2	WWs S	IMP	6	8	33.33	Synergism
			SAM	11	15	36.36	Synergism
			MEM	6	8	33.33	Synergism
			CAZ	6	7	16.67	Additive
			ATM	12	13	8.33	Additive
			FEP	13	12	-7.69	Antagonism
			CN	16	16	0	Indifference
			DOR	6	7	16.67	Additive
			CIP	6	7	16.67	Additive

			AK	20	22	10	Additive
39	19042CNE3	WWs S	IMP	6	9	50	Synergism
			SAM	11	13	18.18	Additive
			MEM	6	7	16.67	Additive
			CAZ	6	6	0	Indifference
			ATM	13	12	-7.69	Antagonism
			FEP	12	8	-33.33	Antagonism
			CN	9	9	0	Indifference
			DOR	6	9	50	Synergism
			CIP	6	9	50	Synergism
			AK	12	13	8.33	Additive
40	19013ONE1	WWs S	IMP	8	8	0	Indifference
			SAM	6	11	83.33	Synergism
			MEM	7	8	14.29	Additive
			CAZ	6	7	16.67	Additive
			ATM	9	8	-11.11	Antagonism
			FEP	10	9	-10	Antagonism
			CN	6	10	66.67	Synergism
			DOR	7	10	42.86	Synergism
			CIP	6	8	33.33	Synergism
			AK	6	8	33.33	Synergism
41	20031ONE5	WWs S	IMP	6	8	33.33	Synergism
			SAM	10	12	20	Synergism
			MEM	6	8	33.33	Synergism
			CAZ	6	8	33.33	Synergism
			ATM	12	11	-8.33	Antagonism
			FEP	6	8	33.33	Synergism
			CN	6	8	33.33	Synergism
			DOR	6	8	33.33	Synergism
			CIP	6	8	33.33	Synergism
			AK	6	8	33.33	Synergism
42	19014CNE2	WWs S	IMP	6	9	50	Synergism
			SAM	14	15	7.14	Additive
			MEM	6	9	50	Synergism
			CAZ	14	14	0	Indifference
			ATM	12	16	33.33	Synergism
			FEP	10	11	10	Additive
			CN	6	7	16.67	Additive
			DOR	6	8	33.33	Synergism
			CIP	6	7	16.67	Additive
			AK	6	6	0	Indifference
43	22019CNE5	WWs S	IMP	30	34	13.33	Additive
			SAM	26	25	-3.85	Antagonism
			MEM	24	26	8.33	Additive
			CAZ	24	20	-16.67	Antagonism
			ATM	16	17	6.25	Additive
			FEP	24	28	16.67	Additive
			CN	17	17	0	Indifference
			DOR	26	26	0	Indifference
			CIP	28	28	0	Indifference
			AK	15	17	13.33	Additive
6	19012ENE2	WWs S	IMP	13	11	-15.39	Antagonism
			SAM	12	15	25	Synergism
			MEM	10	11	10	Additive
			CAZ	6	8	33.33	Synergism

			ATM	11	9	-18.18	Antagonism
			FEP	6	6	0	Indifference
			CN	6	7	16.67	Additive
			DOR	6	8	33.33	Synergism
			CIP	6	7	16.67	Additive
			AK	6	7	16.67	Additive
8	21010ONE3	WWs S	IMP	6	9	50	Synergism
			SAM	6	10	66.67	Synergism
			MEM	6	9	50	Synergism
			CAZ	6	9	50	Synergism
			ATM	13	9	-30.77	Antagonism
			FEP	6	8	33.33	Synergism
			CN	6	7	16.67	Additive
			DOR	6	8	33.33	Synergism
			CIP	6	7	16.67	Additive
			AK	6	7	16.67	Additive
5	19045ENE5	SWs S	IMP	34	32	-5.88	Antagonism
			SAM	28	30	7.14	Additive
			MEM	30	32	6.67	Additive
			CAZ	24	24	0	Indifference
			ATM	20	20	0	Indifference
			FEP	26	26	0	Indifference
			CN	20	22	10	Additive
			DOR	26	27	3.85	Additive
			CIP	30	30	0	Indifference
			AK	18	22	22.22	Synergism
7	21012CNE5	SWs S	IMP	6	9	50	Synergism
			SAM	6	10	66.67	Synergism
			MEM	6	10	66.67	Synergism
			CAZ	6	8	33.33	Synergism
			ATM	6	8	33.33	Synergism
			FEP	6	8	33.33	Synergism
			CN	25	25	0	Indifference
			DOR	6	7	16.67	Additive
			CIP	6	7	16.67	Additive
			AK	9	12	33.33	Synergism
12	19060ENE2	SWs S	IMP	28	36	28.57	Synergism
			SAM	28	28	0	Indifference
			MEM	30	30	0	Indifference
			CAZ	24	28	16.67	Additive
			ATM	23	25	8.70	Additive
			FEP	26	32	23.08	Synergism
			CN	20	20	0	Indifference
			DOR	25	30	20	Synergism
			CIP	29	30	3.45	Additive
			AK	20	20	0	Indifference
13	20070ENE4	SWs S	IMP	24	25	4.17	Additive
			SAM	14	16	14.29	Additive
			MEM	20	21	5	Additive
			CAZ	11	11	0	Indifference
			ATM	12	16	33.33	Synergism
			FEP	14	16	14.29	Additive
			CN	6	6	0	Indifference
			DOR	14	16	14.29	Additive
			CIP	6	8	33.33	Synergism
			AK	8	10	25	Synergism

20	19059ENE1	SWs S	IMP	30	32	6.67	Additive
			SAM	16	22	37.5	Synergism
			MEM	26	24	-7.69	Antagonism
			CAZ	24	26	8.33	Additive
			ATM	21	23	9.52	Additive
			FEP	11	26	136.36	Synergism
			CN	20	22	10	Additive
			DOR	22	25	13.64	Additive
			CIP	28	31	10.71	Additive
			AK	18	20	11.11	Additive
21	20069ENE1	SWs S	IMP	7	9	28.57	Synergism
			SAM	6	12	100	Synergism
			MEM	6	9	50	Synergism
			CAZ	6	10	66.67	Synergism
			ATM	12	10	-16.67	Antagonism
			FEP	6	10	66.67	Synergism
			CN	6	7	16.67	Additive
			DOR	6	10	66.67	Synergism
			CIP	6	8	33.33	Synergism
			AK	6	7	16.67	Additive
16	19022CNE4	WWs C-W	IMP	6	8	33.33333	Synergism
			SAM	13	15	15.38462	Additive
			MEM	6	8	33.33333	Synergism
			CAZ	6	7	16.66667	Additive
			ATM	14	14	0	Indifference
			FEP	6	7	16.66667	Additive
			CN	6	8	33.33333	Synergism
			DOR	6	7	16.66667	Additive
			CIP	6	7	16.66667	Additive
			AK	6	9	50	Synergism
17	22022CA5	WWs C-W	IMP	32	32	0	Indifference
			SAM	28	32	14.29	Additive
			MEM	30	32	6.67	Additive
			CAZ	26	36	38.46	Synergism
			ATM	20	21	5	Additive
			FEP	28	24	-14.29	Antagonism
			CN	20	21	5	Additive
			DOR	28	30	7.14	Additive
			CIP	30	33	10	Additive
			AK	19	21	10.53	Additive
25	21001CNE5	WWs C-W	IMP	6	7	16.67	Additive
			SAM	17	20	17.65	Additive
			MEM	6	7	16.67	Additive
			CAZ	11	13	18.18	Additive
			ATM	15	19	26.67	Synergism
			FEP	15	16	6.67	Additive
			CN	6	7	16.67	Additive
			DOR	6	7	16.67	Additive
			CIP	6	6	0	Indifference
			AK	6	6	0	Indifference
28	22007CA4	WWs C-W	IMP	36	40	11.11	Additive
			SAM	34	34	0	Indifference
			MEM	32	32	0	Indifference
			CAZ	30	30	0	Indifference
			ATM	22	24	9.09	Additive

			FEP	32	34	6.25	Additive
			CN	25	25	0	Indifference
			DOR	32	32	0	Indifference
			CIP	32	35	9.38	Additive
			AK	23	24	4.35	Additive
15	20113ENE3	SWs C-W	IMP	9	10	11.11	Additive
			SAM	6	15	150	Synergism
			MEM	6	10	66.67	Synergism
			CAZ	6	7	16.67	Additive
			ATM	10	12	20	Synergism
			FEP	11	12	9.09	Additive
			CN	6	7	16.67	Additive
			DOR	6	7	16.67	Additive
			CIP	6	8	33.33	Synergism
			AK	6	7	16.67	Additive
23	19051ENE2	SWs C-W	IMP	32	34	6.25	Additive
			SAM	28	30	7.14	Additive
			MEM	22	28	27.27	Synergism
			CAZ	22	26	18.18	Additive
			ATM	20	22.24	11.2	Additive
			FEP	26	28	7.69	Additive
			CN	19	21	10.53	Additive
			DOR	24	29	20.83	Synergism
			CIP	30	32	6.67	Additive
			AK	18	21	16.67	Additive
24	22006CA3	SWs C-W	IMP	30	34	13.33	Additive
			SAM	28	32	14.29	Additive
			MEM	30	32	6.67	Additive
			CAZ	26	28	7.69	Additive
			ATM	21	21	0	Indifference
			FEP	24	30	25	Synergism
			CN	23	22	-4.35	Antagonism
			DOR	25	36	44	Synergism
			CIP	28	32	14.29	Additive
			AK	19	22	15.79	Additive
26	20056CNE2	SWs C-W	IMP	6	9	50	Synergism
			SAM	14	13	-7.14	Antagonism
			MEM	6	8	33.33	Synergism
			CAZ	6	6	0	Indifference
			ATM	15	14	-6.67	Antagonism
			FEP	10	11	10	Additive
			CN	6	7	16.67	Additive
			DOR	6	8	33.33	Synergism
			CIP	6	11	83.33	Synergism
			AK	6	6	0	Indifference
	P. aeruginosa ATCC 27853		IMP	24	23	-4.17	Antagonism
			TZP	28	30	7.14	Additive
			ATM	27	26	-3.70	Antagonism
			FEP	30	28	-6.67	Antagonism
			CAZ	28	28	0	Indifference
			AK	23	23	0	Indifference
			CN	20	20	0	Indifference
			DOR	33	30	-9.09	Antagonism
			CIP	33	32	-3.03	Antagonism
			MEM	6	7	16.67	Additive

IMP (imipenem), 10 µg; TZP (piperacillin-tazobactam), 85 µg; ATM (aztreonam), 30 µg; FEP (cefepime), 30 µg; CAZ (ceftazidime), 30 µg; MEM (meropenem), 10 µg; CIP (ciprofloxacin), 5 µg; SAM (ampicillin-sulbactam), 20 µg; AK (amikacin), 30 µg; DOR (doripenem), 10 µg; CN (gentamicin), 10 µg; %, percentage; IZD, inhibition zone diameter.
